# Dimensional Engineering of Co/W Superlattices Enables Out-of-Plane Spin Polarization with 90% Field-Free Switching in Perpendicular CoFeB

**DOI:** 10.1021/acs.nanolett.6c04408

**Published:** 2026-10-12

**Authors:** Yu-Lon Lin, Jing-Wen Chen, Yan-Zhen Huang, Tzu-Chuan Hsin, Chih-Wei Cheng, Yu-Han Huang, Ssu-Yen Huang, Shintaro Hara, Tatsuya Kishi, Yuan-Chieh Tseng

**Affiliations:** † Department of Materials Science & Engineering, 34914 National Yang-Ming Chiao-Tung University, Hsinchu 30010, Taiwan, ROC; ‡ Department of Materials Science & Engineering, 33561 National Taiwan University, Taipei 10617, Taiwan, ROC; § Taiwan Semiconductor Research Institute, Hsinchu 30010, Taiwan, ROC; ∥ Department of Physics, National Taiwan University, Taipei 10617, Taiwan, ROC; ⊥ Center of Atomic Initiatives for New Materials, National Taiwan University, Taipei 10617, Taiwan, ROC; # TopoLogic Inc., Tokyo 113-0033, Japan

**Keywords:** spin−orbit torque, field-free switching, composite SOT layer, CoFeB

## Abstract

Deterministic spin–orbit torque (SOT) switching of perpendicular magnets without external magnetic fields remains a central challenge for scalable SOT-MRAM. Here, we demonstrate that dimensional engineering of a Co/W superlattice spin–orbit layer enables >90% field-free switching of perpendicular CoFeB. Harmonic Hall measurements reveal an unconventional out-of-plane torque, while first-principles calculations reveal reconstruction of the spin-resolved electronic structure and increased W-projected out-of-plane spin character, together supporting the modification of torque symmetry through Co/W superlattice engineering. The optimized superlattice achieves a critical current density of 23.3 MA cm^–2^ and a thermal stability factor Δ ≈ 45. Nanoscale devices (100 nm) preserve deterministic field-free reversal with comparable efficiency, confirming scalability toward magnetic tunnel junction integration. Micromagnetic analysis shows that out-of-plane spin-polarized torque together with canted anisotropy enables intrinsic symmetry breaking, highlighting superlattice engineering as a route to tailor torque symmetry for effective field-free SOT devices.

## Graphical abstract

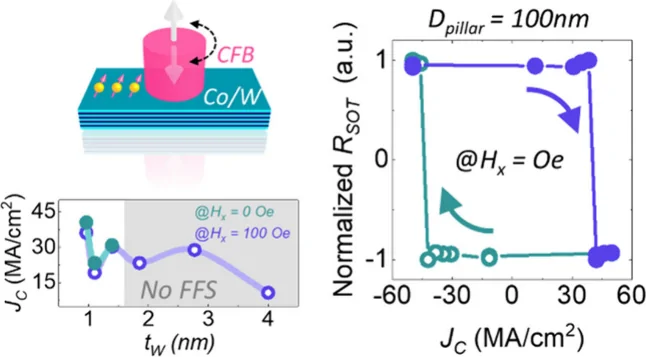

Spin–orbit torque magnetic random-access memory (SOT-MRAM) is a leading candidate for next-generation nonvolatile memory, offering ultrafast operation, superior endurance, and a robust three-terminal architecture that separates read and write paths to suppress read disturbance and enhance reliability.^1–5^ Yet, despite these strengths, SOT-MRAM remains distant from large-scale commercialization. The barrier is not architectural but physical: in perpendicular magnetic anisotropy (PMA) devices, i.e., type-Z configurations, deterministic SOT switching requires an external magnetic field,^2–4^ a constraint that negates the energy efficiency and scalability essential for industrial adoption. Numerous field-free schemes have been proposed, including in-plane electric field,^6,7^ bending current,^8^ antiferromagnetic (AFM) exchange coupling,^9–11^ structural asymmetry,^12–14^ and unconventional spin sources such as Weyl semimetals and noncollinear antiferromagnets,^15–19^ as well as magnetic hard-mask designs.^20^ However, these concepts often demand complex fabrication or introduce integration barriers inconsistent with high-density, CMOS-compatible manufacturing. Composite SOT stacks combining an in-plane ferromagnet with a normal metal meet this criterion but suffer from current shunting and parasitic torques that degrade write fidelity and reliability.^21–26^ Moreover, despite these limitations, composite SOT layers with tailored low-dimensional heavy-metal multilayer architectures have enabled high spin Hall conductivity and field-free magnetization switching, though only in type-Y SOT configurations.^27^ Multilayers and alloy-based SOT systems have shown robust field-free switching (FFS) across various device geometries,^28–30^ yet none simultaneously achieve the two prerequisites for scalable memory: seamless integration with CoFeB/MgO for high tunnel magnetoresistance and the generation of out-of-plane spin current required for type-Z reversal. This unresolved trade-off defines the central gap separating proof-of-concept SOT devices from a truly manufacturable, field-free SOT-MRAM technology.

In this work, we demonstrate all-electrical field-free magnetization switching in a type-Z Co/W/CoFeB/MgO SOT structure and identify unconventional torque components. Systematic engineering of Co/W repetition, Co concentration, and W sublayer thickness reveals that field-free switching strongly depends on the dimensional configuration of the spin–orbit layer. First-principles calculations show a configuration-dependent reconstruction of the spin-resolved electronic structure, with enhanced W-projected out-of-plane spin character in the periodically modulated Co/W structure. Together with the experimentally observed unconventional torque components, these calculations indicate that Co/W dimensional modulation modifies the local symmetry constraints of the W-derived electronic states, providing microscopic electronic-structure support for the resulting modification of torque symmetry.

The optimized Co/W device achieves deterministic field-free SOT switching with a switching ratio exceeding 90% at a low switching current density (J_c_) of 23.3 MA cm^–2^ and a thermal stability factor of 44.64. Importantly, scaling to a 100 nm nanopillar preserves the field-free switching, with a switching ratio above 90% and J_c_ of 51 MA cm^–2^. These results establish dimensional engineering of Co/W superlattices as an effective strategy for generating unconventional out-of-plane spin polarization and enabling efficient field-free SOT switching, providing a new design pathway for scaled SOT devices.

All films were deposited on Si/SiO_2_ substrates by ultrahigh-vacuum (UHV) magnetron sputtering (base pressure <5.0 × 10^–9^ Torr) at room temperature. Co, CoFeB, and MgO layers were sputtered at 50 W, while W, Ta, and Pt were sputtered at 30 W. To enhance perpendicular magnetic anisotropy, the films were annealed at 250 °C, followed by photolithography and Ar ion milling to define Hall-bar devices. The width of the current channel in these devices was 10 μm.

The fabrication of nanopillar devices involved a multistep process combining lithography and etching techniques. Initially, a hard mask comprising a 10 nm Pt layer atop a 30 nm W layer was deposited. This mask was then patterned using electron-beam lithography (Raith Voyager), and the pattern was transferred into the underlying layers via a sulfur hexafluoride (SF6)-based reactive ion etching process. Subsequently, the CoFeB/MgO was defined into pillar structures using a two-step angled Ar ion milling technique. The final steps involved defining the SOT channel and forming the electrical contacts. This was achieved through a combination of standard photolithography and subsequent metallization. Electrical transport was measured using a Keithley 6221 current source and a 2182A nanovoltmeter. The magnetization state of the CoFeB pillar was monitored after each pulse injection by Hall resistance measurements with a 200 μA DC bias. The writing pulse was set to be 500 μs in the field-dependent SOT switching measurement.

The proposed Co/W superlattice SOT structure, illustrated in Figure 1a, was fabricated by depositing a [W/Co] spin–orbit (SO) multilayer, consistently terminated with a W layer, via ultrahigh vacuum (UHV) sputtering on a Si substrate with 200 nm of thermally grown SiO_2_. A perpendicularly magnetized CoFeB (1.2 nm) layer was subsequently deposited atop the Co/W multilayers for current-induced magnetization switching characterization. Finally, a MgO (1.5 nm)/Ta (2 nm) capping layer was added to prevent oxidation. We introduce a two-digit footnote notation to encode the structural and compositional parameters of each sample. Taking Co/W1-8 as an example (Figure 1b), the first digit (1) represents the number of [W/Co] repetition in the SO stack. Each stack is designed to terminate with a W sublayer on both top and bottom sides, and the total SO stack is fixed at 6 nm. Consequently, varying the repetition number within this constant thickness directly modulates the Co concentration across the stack. The second digit (8) quantifies this Co concentration, as determined by energy-dispersive X-ray spectroscopy (EDX) in transmission electron microscopy (TEM) scans across the Co/W superlattice. This notation provides a consistent way to describe the structural and compositional parameters of all samples in this study.

**Figure 1:**
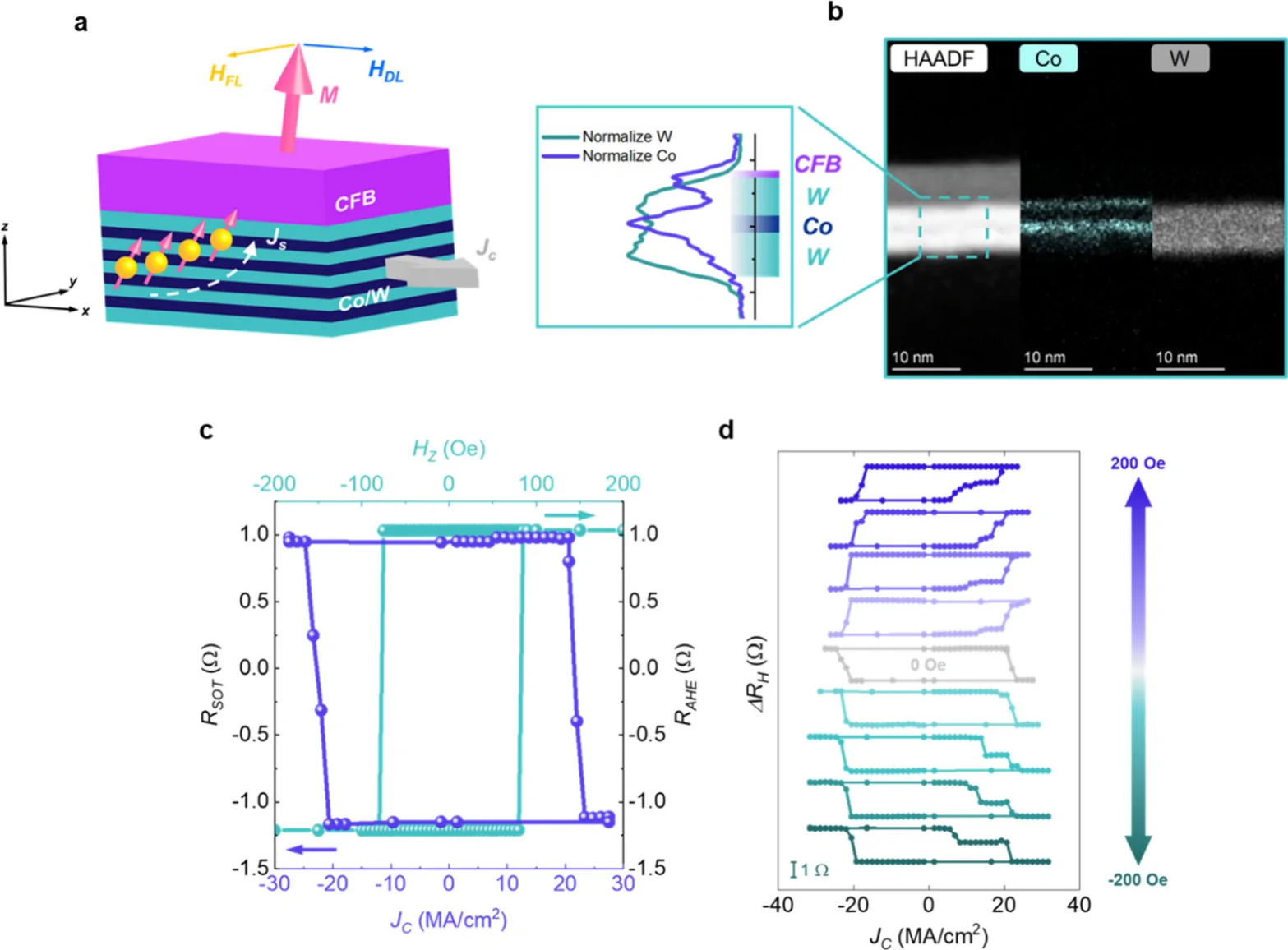
Field-free current-induced magnetization switching in a Co/W superlattice SOT device. (a) A schematic illustration of the fabricated Co/W superlattice SOT device. The pink arrow represents the magnetization direction of the CFB free layer. In the Co/W SO layer, the gray arrow indicates the direction of the applied current, while the small pink arrows illustrate the generation and flow of spins toward the *z*-direction via the SHE from the Co/W SOT channel. *H*_*DL*_ and *H*_*FL*_ refer to the damping-like and field-like torques, respectively. The schematic illustrates the central physical mechanism underlying the proposed structure in this work. (b) TEM EDX-mapping of the Co/W1-8 device to illustrate the sandwich/superlattice structure. The normalized EDX line profiles of Co and W, together with the accompanying schematic, are provided as a guide to the eye to illustrate the multilayer structure. (c) The AHE loop of the Co/W4-8 SOT device, which clearly demonstrates the PMA nature. The current-induced magnetization switching exhibits field-free magnetization switching, achieving a field-free switching ratio (R_AHE_/R_SOT_) as high as 90%. (d) Current-induced magnetization switching of the Co/W4-8 device measured under external magnetic fields ranging from +200 Oe to −200 Oe.

Under this Co/W stacking scheme, all samples exhibited perpendicular magnetic anisotropy (PMA), verified by the anomalous Hall effect (AHE) measurement, as shown in Figure 1c. We found that with the Co/W4–8 condition, i.e., when the [Co/W] bilayer with a total thickness of 6 nm was repeated four times, a high field-free magnetization switching ratio (Δ*R*_*SOT*_/Δ*R*_*AHE*_) of approximately 90% was achieved. Here, we defined the Δ*R*_*SOT*_ = [|*R*_*H*_^+^ (+*I*_*write*_ → +0)| – |*R*_*H*_^–^ (−*I*_*write*_ → −0)|] and Δ*R*_*AHE*_ = [|*R*_*H*_^+^ (+*H* → +0)| – |*R*_*H*_^–^ (−*H* → −0)|], respectively. The *R*_*H*_^+^ (*R*_*H*_^–^) denotes the difference in Hall resistance by current-induced (or field-induced) switching at zero write current (or zero field). Figure 1d displays representative SOT switching loops for the [Co/W]_4_ structure under various in-plane magnetic fields (*H*_*x*_). The resistivities of W, Co/W, and CoFeB were determined to be 185, 175, and 170 μΩ cm, respectively, yielding an estimated switching current density of 23.3 MA cm^–2^ after accounting for current shunting effects. These results indicate that the Co/W superlattice, when engineered within the PMA-SOT framework, provides an effective platform for achieving robust field-free magnetization switching. Motivated by this observation, the subsequent sections examine how the Co/W superlattice configuration influences the magnetic properties and SOT characteristics associated with the observed field-free switching.

For comparison, a reference sample incorporating a conventional W-alone SOT stack was fabricated (Supplementary S1). A 4 nm-thick W layer was chosen for its optimal charge-to-spin conversion efficiency,^31,32^ while further thickness increase promotes the formation of the α-phase, thereby reducing spin conversion efficiency.^33,34^ Unlike the Co/W4–8 case, the W reference sample required an external magnetic field to achieve magnetization reversal. This behavior is consistent with the conventional SOT symmetry of W, in which the dominant y-polarized spin component alone is insufficient to break the symmetry required for deterministic switching of a perpendicular magnet.

Field-dependent current-induced magnetization switching measurements were systematically performed on the superlattice structure with varying [Co/W] repetition numbers, while maintaining a constant total SOT-layer thickness of 6 nm. Figure 2a illustrates the multilayer stacking structures of the reference W (denoted as W(4)), and [Co/W] SOT devices with variable superlattice repetition and Co concentration (denoted as Co/W1-8, Co/W4-8, and Co/W4-24). Note that both Co/W4-8 and Co/W4-24 involve four repetitions of the [Co/W] superlattice. However, in Co/W4-24, we intentionally increased the Co thickness to investigate how, under a fixed superlattice repetition number, this adjustment affects field-free switching. Please refer to Supplementary 2 for additional details on the layer thicknesses of all the investigated [Co/W] samples. The corresponding switching phase diagrams (SPDs), summarized in Figure 2b–e for reference W, Co/W1-8, Co/W4-8, and Co/W4-24 samples, plot the J_c_ as a function of H_*x*_. Notably, field-free magnetization switching emerges in the Co/W4-8 and Co/W4-24 samples, in sharp contrast to the absence of such behavior in devices with only one or two Co repetitions (Supplementary S3, S4).

**Figure 2:**
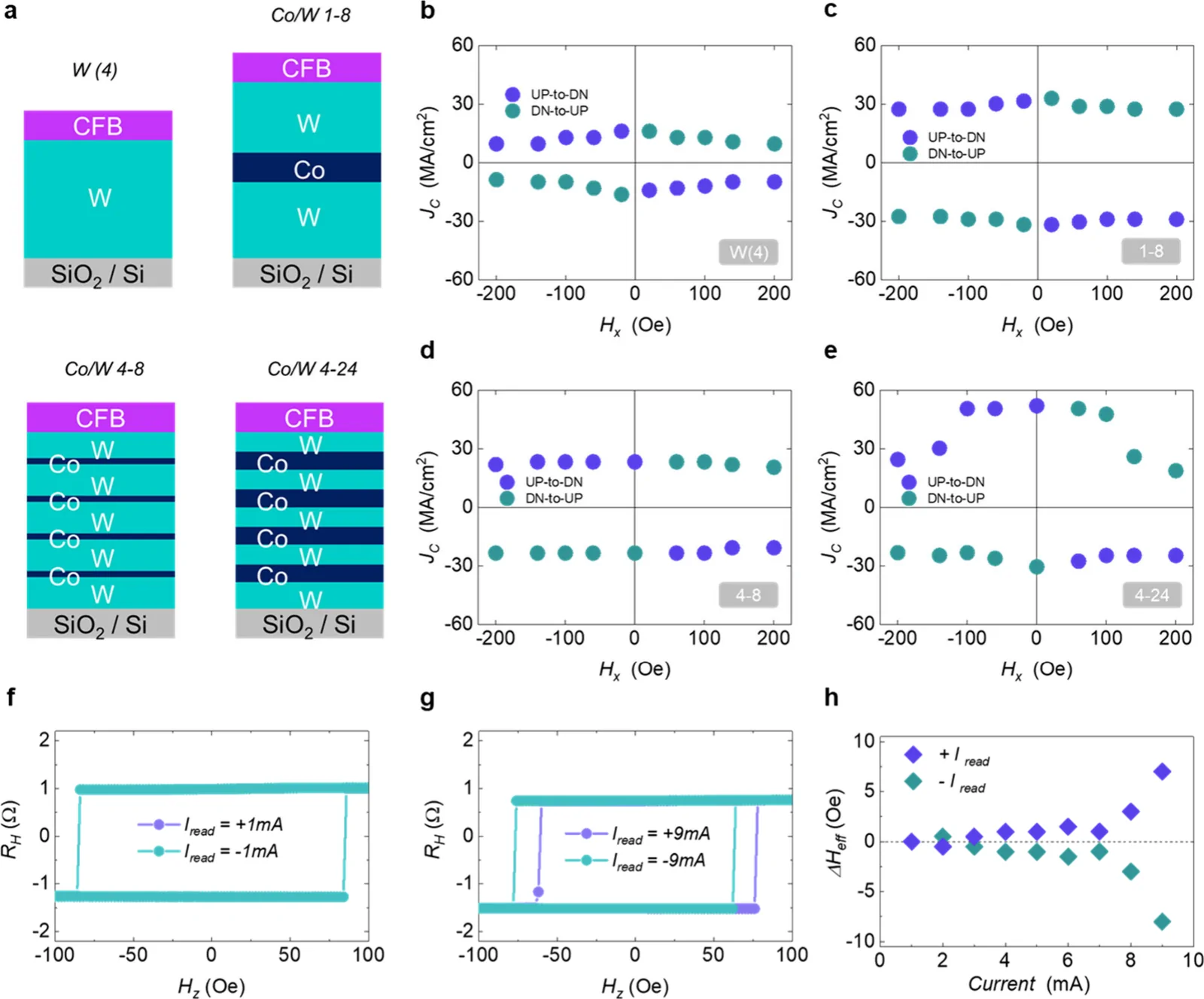
Current-induced magnetization switching phase diagrams and AHE loop-shift characterization of Co/W SOT devices. (a) Structural schematics of the W control sample and the Co/W superlattice, highlighting the layer stacking of Co/W1-8, Co/W4-8, and Co/W4-24 devices. (b–e) Switching phase diagrams for (b) pure W, and Co/W SOT devices of (c) Co/W1-8, (d) Co/W4-8, and (e) Co/W4-24, respectively. (f, g) Current-induced AHE loop-shift measurements for a representative Co/W4–8 SOT device under read current amplitudes of 1 mA and 9 mA, respectively. (h) Extracted current-induced SOT effective field (*H*_eff_) as a function of read current, summarizing the AHE loop-shift results of the Co/W4–8 SOT.

The evolution of the SPDs indicates a pronounced modification of the switching characteristics with Co/W superlattice modulation. For the W (Figure 2b) and Co/W1-8 (Figure 2c) devices, J_c_ increases steeply as the in-plane field decreases, characteristic of conventional damping-like torque-dominated switching driven by y-polarized spins originating from the heavy metal layer.^4^ In contrast, Co/W4-8 and Co/W4-24 (Figure 2d,e) exhibit markedly different behaviors: the SPD of Co/W4-8 is nearly field-independent, yielding a nearly flat J_c_-*H*_*x*_ response, whereas Co/W4-24 develops pronounced asymmetry and field shifts. The nearly field-independent SPD of Co/W4-8 suggests the presence of an additional symmetry-breaking torque that substantially reduces the dependence of magnetization reversal on the externally applied in-plane field. As discussed below, this interpretation is supported by the AHE loop-shift and second-harmonic Hall measurements, which reveal a finite out-of-plane torque component. Such an unconventional torque provides an intrinsic source of symmetry breaking, thereby enabling magnetization reversal with substantially reduced dependence on an external symmetry-breaking field.

To verify the presence of this unconventional spin polarization, we performed AHE loop-shift measurements under different applied currents. As shown in Figure 2f–h, a distinct current-induced AHE loop shift appears in the Co/W4-8 device, revealing a finite current-induced perpendicular effective field consistent with an unconventional out-of-plane torque component. Analyses of the current-induced SOT effective field (H_eff_) as a function of Co concentration and repetition number further reveal that, this out-of-plane torque component is absent in Co/W1-8 and Co/W2-8 samples (Supplementary S5). The magnitude current-induced SOT effective field (*H*_*eff*_) was defined by the equations Δ*H*_*eff*_ (+*I*) = [*H*_*center*_ (+*I*) – *H*_*center*_ (−*I*)]/2 and Δ*H*_*eff*_ (−*I*) = [*H*_*center*_ (−*I*) – *H*_*center*_ (+*I*)]/2, respectively. The absence of measurable *H*_eff_ in these devices indicates that the corresponding out-of-plane torque component, if present, is below our experimental detection limit, consistent with the predominantly conventional heavy-metal-like switching behavior.

The pronounced asymmetry in the SPD of Co/W4-24 (Figure 2e) is consistent with a canted magnetic configuration, which is promoted by the enhanced in-plane anisotropy, in contrast to the Co/W4-8 sample. Increasing the Co concentration slightly tilts the CoFeB easy axis away from the film normal, reducing the energy barrier for magnetization reversal and facilitating efficient current-induced switching. These observations indicate that the switching behavior is governed by the combined effects of the unconventional torque components and the magnetic-anisotropy configuration. Robust field-free magnetization switching is favored when the Co sublayers are confined to the low-dimensional regime and the W sublayer thickness is reduced below approximately 1.5 nm. This dimensional dependence further suggests that both the local Co/W configuration and spin transport through the ultrathin W sublayers play important roles in determining the overall SOT response. Specifically, the thickness of each W sublayer is maintained within the reported spin diffusion length (l_sf_) of W (0.9 nm ≤ l_sf_ ≤ 1.5 nm),^31^ a regime in which spin relaxation across an individual W sublayer is expected to be limited.

To obtain microscopic insight into the influence of Co/W dimensional modulation, we performed first-principles calculations using two model configurations corresponding to the experimentally investigated Co/W1-8 and Co/W4-8 structures (Supplementary S6). Configuration A, corresponding to Co/W1-8, consists of a W/Co/W sandwich structure with a W:Co:W thickness ratio of approximately 25:4:25, whereas Configuration B, corresponding to Co/W4-8, consists of periodically alternating W and Co sublayers with a thickness ratio of approximately 10:1:10:1:10:1:10:1:10. The calculations reveal a configuration-dependent reconstruction of the spin-resolved electronic structure near the Fermi level. In particular, the W-projected band structures in Configuration B show an increased presence of out-of-plane *S*_*z*_^*W*^ character compared with Configuration A. This increase indicates that repeated Co/W dimensional modulation promotes out-of-plane spin character in the W-derived electronic states. The emergence of this out-of-plane spin character can be understood from the symmetry breaking introduced by Co/W dimensional modulation. Previous studies have established that structural and electronic symmetry critically determine the allowed spin-polarization directions, and that reduced symmetry constraints can permit unconventional out-of-plane spin components.^35–37^ In the present Co/W superlattice, periodic insertion of ultrathin Co layers breaks the symmetry of the W environment by creating inequivalent Co/W interfaces and modifying W–W and Co–W hybridization. This asymmetry lifts symmetry constraints on the W-derived states, allowing suppressed orbital components to emerge. Combined with the strong spin–orbit coupling of W, the electronic-state reconstruction provides a basis for the enhanced out-of-plane spin character.

To experimentally resolve the corresponding torque components, we performed second-harmonic Hall measurements, which show finite *ξ*_*DL*__,*x*_ and *ξ*_*DL*__,*z*_, indicating that the Co/W superlattice generates spin-polarization components beyond the conventional in-plane *σ*_*y*_ symmetry (Supplementary S7). The experimentally resolved out-of-plane *ξ*_*DL*__,*z*_ component is consistent with the increased W-projected *S*_*z*_^*W*^ character revealed by the first-principles calculations, further supporting the role of Co/W dimensional modulation in modifying the torque symmetry. Importantly, the optimized Co/W superlattice also retains a large conventional damping-like efficiency (*ξ*_*DL*__,*y*_ = −0.363), comparable to our W reference sample (*ξ*_*DL*__,*y*_ = 0.378), while additionally exhibits unconventional torque components. The out-of-plane component *ξ*_*DL*__,*z*_ corresponds to a current-induced perpendicular effective field consistent with the AHE loop shifts, whereas *ξ*_*DL*__,*x*_ may contribute to the characteristic asymmetry of the SPD. We also considered alternative symmetry-breaking mechanisms, including stray fields, structural asymmetry, and interlayer magnetic coupling. Although minor contributions from these effects cannot be completely excluded, they cannot consistently explain the strong dependence of field-free switching on Co/W dimensional modulation, the AHE loop shifts, the evolution of the SPD, and the finite *ξ*_*DL*__,*z*_ extracted from second-harmonic Hall measurements. Taken together, the calculations and experiments indicate that Co/W superlattice engineering reconstructs the spin-resolved electronic structure and is accompanied by the emergence of experimentally resolvable unconventional torque components, without substantially compromising the conventional damping-like torque.

This behavior is distinct from that reported by Huang et al.^27^ for a related composite-W system with an annealing-induced anisotropic crystalline texture, in which 1.65 nm W sublayers were separated by ultrathin 0.14 nm Co insertion layers primarily to stabilize the β-W phase. Their harmonic Hall analysis revealed a finite x-polarized damping-like component for the device orientation with current perpendicular to the annealing direction, whereas the extracted out-of-plane component remained approximately zero. In contrast, the optimized Co/W superlattice in the present work employs thinner W sublayers (t_W_ ≈ 1.1 nm) and exhibits a clearly resolved *ξ*_*DL*__,*z*_ component. These differences, together with the calculated sensitivity of the spin-resolved electronic structure to Co/W dimensional modulation, highlight the importance of the specific dimensional configuration in influencing the unconventional torque symmetry of Co/W-based systems.

Figures 3a and 3b summarize J_c_ as a function of the individual W sublayer thickness (t_W_) within the Co/W framework under H_*x*_ = 100 and 0 Oe, respectively. The data points presented correspond to the sample conditions described in Supplementary 2. An optimal regime for field-free current-induced switching emerges when t_W_ is around 1.1 nm, with an achieved switching ratio around 93%. Further increasing t_W_ progressively suppresses the field-free switching, with the switching ratio decreasing to 49% in the Co/W3–8 device, indicating a gradual recovery toward predominantly conventional W-like SOT behavior. Overall, these results show that SOT switching characteristics are highly sensitive to the dimensional configuration of the Co/W multilayer, particularly the thickness of the individual W sublayer.

**Figure 3:**
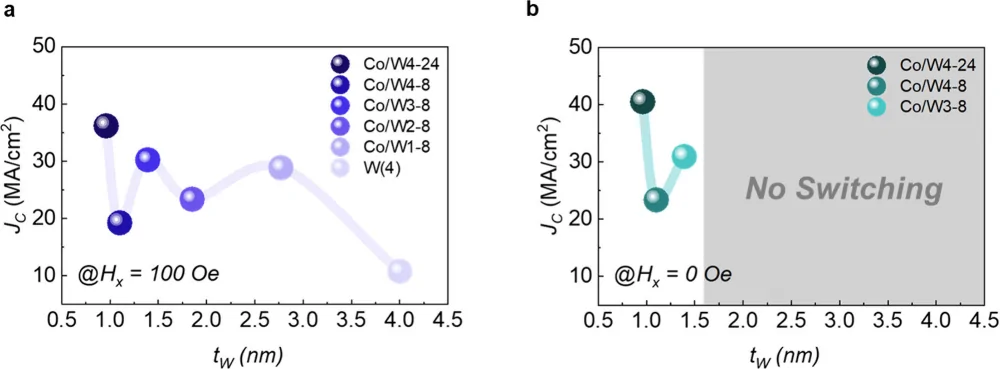
Summary of SOT performance as a function of *t*_*W*_. Switching current density under (a) field-assist and (b) field-free conditions as a function of the thickness of W sublayers. The numbers in parentheses denote the switching ratio for each field-free device.

We have performed macrospin and micromagnetic simulations to investigate the dependence of the J_c_ and SOT-induced magnetization on noncollinear spin moments (Supplementary S8). The simulations reveal that the coupling between the PMA and in-plane magnetic anisotropy (IMA) layers leads to a deflection of the magnetic easy axis. When a current is applied along the IMA easy axis, the PMA layer exhibits a pronounced shift in its magnetization reversal curve, consistent with the experimental results of the Co/W4–24 sample.

We further considered the configuration in which the easy axis of the PMA layer is tilted away from the *z*-axis toward the *y*-axis, as depicted in Figure 4a. Unlike the *x*-axis tilting scenario, a tilting toward the *y*-axis does not produce a shift in the current-induced reversal curve. Instead, it enables deterministic, field-free magnetization switching through SOT, owing to symmetry breaking in the spin polarization direction. Moreover, upon applying an in-plane magnetic field H_*x*_, the system retains a key characteristic of the untilted configuration; namely, a reduction in J_c_ with increasing field strength. SOT phase diagram on the Co/W4–24 sample in Figure 2e also indicated that increasing the Co concentration in the underlayer introduces a horizontal magnetic component, aligning with the observed eas*y*-axis deflection in the PMA layer.

**Figure 4:**
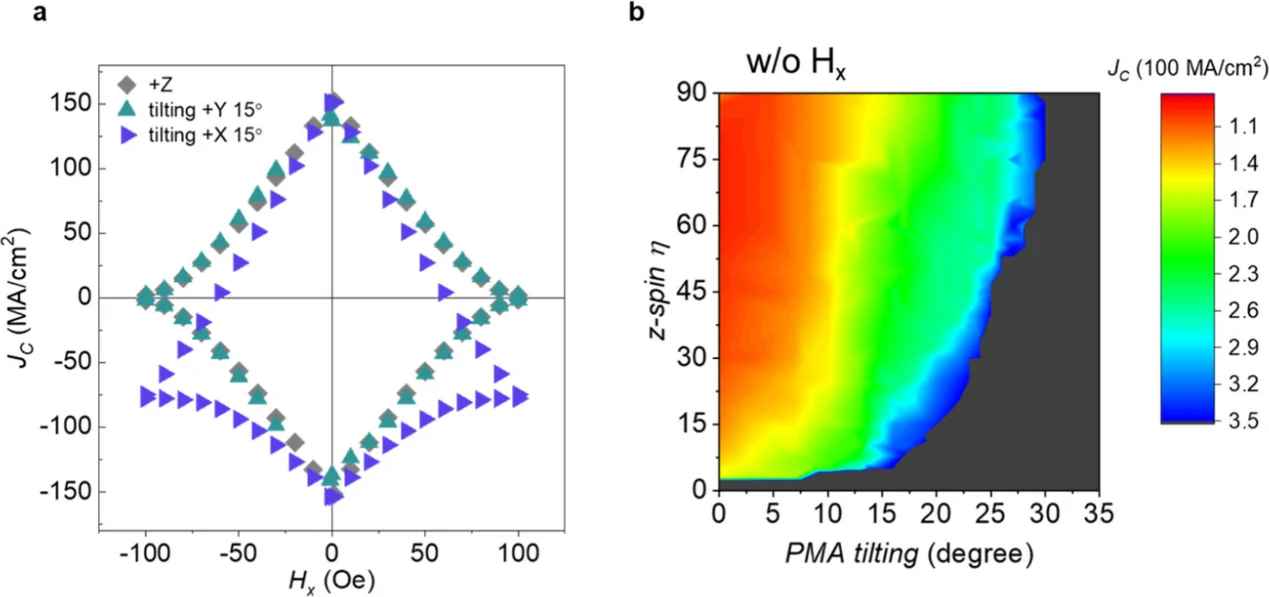
SOT-induced magnetization on noncollinear spin moments. (a) Simulated SPD for three magnetization configurations of the PMA layer: out-of-plane (+Z, black circles), tilted + Y 15° (green triangles), and tilted + X 15° (purple triangles). (b) The simulated phase diagram illustrates how *J*_*c*_ (measured in 100 MA cm^–2^) depends on the PMA tilting angle (tilting from the z toward the *x*-axis) and the initial out-of-plane spin component (z-spin) in the absence of an external magnetic field (*H*_*x*_ = 0).

Figure 4b presents the simulated SPD of field-free magnetization reversal, depicting J_c_ as a function of the PMA tilting angle and the initial z-spin component. The spin polarization direction is defined as σ = (0, cosη, sinη), where η denotes the spin polarization angle that sets the magnitude of the z-spin component. The simulations reveal a competitive relationship between the z-spin component and PMA tilting: increasing the z-spin component substantially reduces J_c_, whereas a larger PMA tilting suppresses current-induced reversal. Field-free switching fails when the out-of-plane torque from the z-polarized spin current is insufficient to overcome the enhanced damping-like precession—analogous to type-Z or type-X dynamics—induced by the tilted anisotropy. The resulting heat map captures the interplay between the z-polarized torque and the PMA tilting, with the color scale ranging from red (low J_c_) to blue (high J_c_), and finally to gray, which designates regions where field-free switching is not achieved. These simulations demonstrate that the interplay between a z-polarized spin component and canted magnetic anisotropy can enable robust field-free switching, consistent with the experimentally observed switching behavior and unconventional torque components. Another potential field-free switching mechanism is the orange-peel effect (OPE),^38,39^ which arises from the interfacial roughness of the underlying heavy metal layer. Atomic force microscopy (AFM) was employed to characterize the fabricated Co/W1-8 and Co/W4–8 samples (Supplementary S9). Given the atomically flat surface morphology observed in our Co/W system, contributions from OPE can be ruled out.

Finally, the optimal Co/W4–8 SOT structure was miniaturized from a Hall bar to the nanometer scale to evaluate its current-induced magnetization switching behavior with respect to the scaling effect. Figure 5a shows the schematic of the Co/W4–8 device configured with a 100 nm magnetic nanopillar, a geometry dimensionally compatible with magnetic tunnel junction (MTJ) integration. The device exhibits a good PMA and robust field-free electrical switching, as illustrated in Figure 5b, confirming that the symmetry-breaking mechanism remains effective even at the nanoscale. Notably, the 100 nm nanopillar displays enhanced coercivity compared to the Hall-bar structures (Supplementary S10), suggesting improved thermal stability in confined geometries

**Figure 5:**
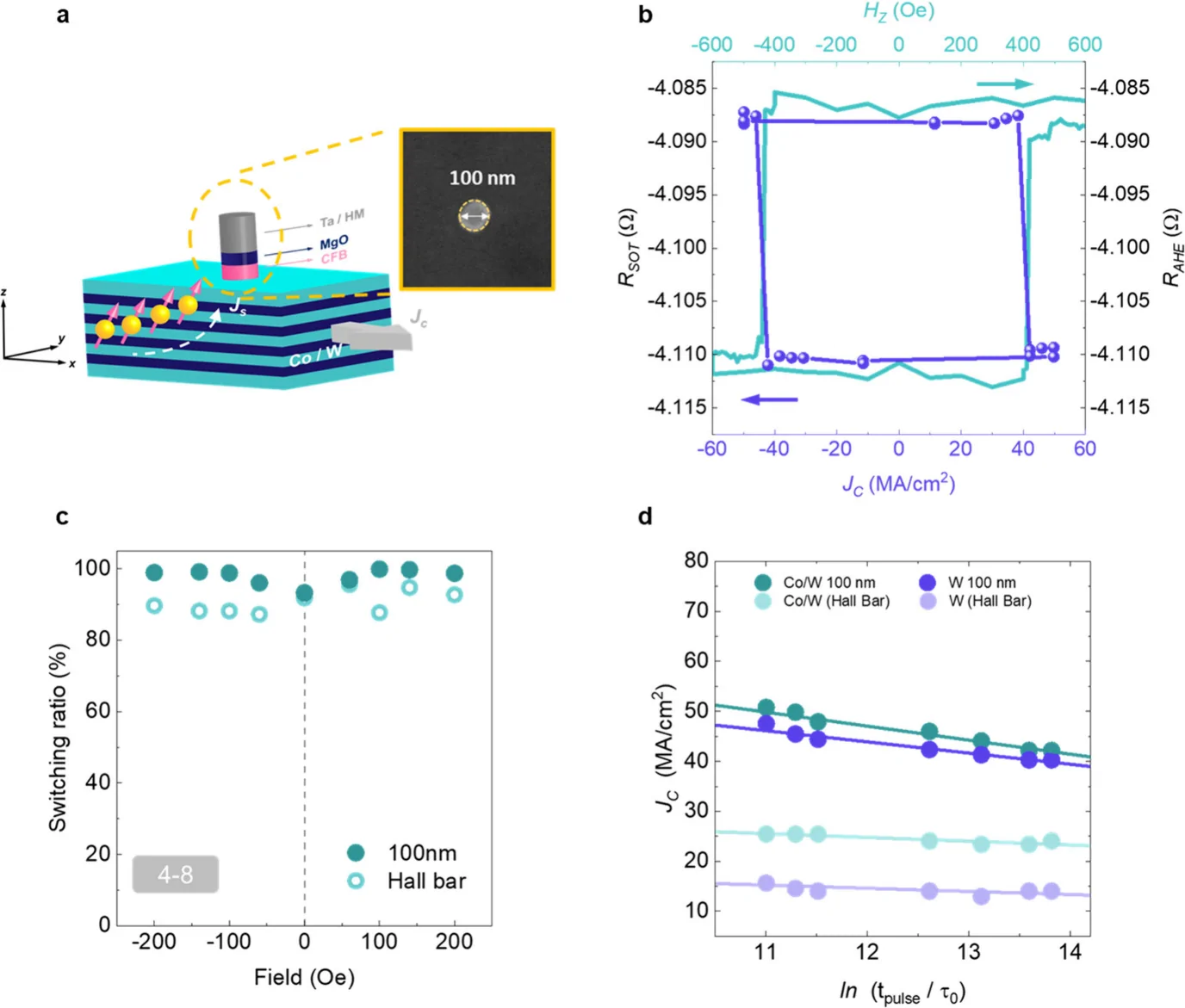
Current-induced magnetization switching in a Co/W SOT nanopillar structure. (a) Schematic illustration of a Co/W4-8 SOT nanopillar structure. The magnetic pillar is etched and patterned directly onto the Co/W SOT channel. The inset shows the SEM image of a nanopillar scaled down to 100 nm. (b) Demonstration of PMA and field-free magnetization switching in a 100 nm diameter Co/W nanopillar device. (c) Switching ratio comparison for Co/W nanopillar devices with critical dimensions of 100 nm and 10 μm (Hall-bar). (d) Critical switching current density as a function of pulse width for a Hall bar and a nanopillar device. The performance of Co/W4-8 and conventional W SOT channels is compared.

As shown in Figure 5c, both the nanopillar and Hall-bar Co/W4-8 SOT devices achieve a large field-free switching ratio of up to 90%, underscoring the high efficiency of the Co/W composite layer. The switching ratio remaining below full reversal (i.e., 100%) is attributed to domain wall-mediated switching dynamics and pinning effects inherent to the nanopillar geometry, rather than insufficient SOT generation. Further optimization of the etching process and device geometry is expected to reduce pinning sites and enhance switching uniformity. Pulse-width-dependent measurements (Supplementary S11) further reveal that, while the 100 nm reference W device requires a 60 Oe in-plane bias field for deterministic switching, the Co/W device maintains fully field-free operation. Figure 5d summarizes the pulse-width dependence of the J_c_ for both device geometries. The increase in J_c_ with decreasing pillar size is likely attributed to single-domain formation within the confined structure.

The thermal stability factor (Δ) and intrinsic critical switching current density (J_c0_) were extracted using the thermal activation model of SOT switching:^40^

1 $$J_{c}=J_{c0}\left[1-\frac{1}{\Delta }\ln\left(\frac{t_{pulse}}{\tau_{0}}\right)\right]$$

The fitted parameters yield Δ values of 28.78 for Co/W4–8 (100 nm nanopillar), 44.64 for Co/W4–8 (Hall device), 31.63 for W (100 nm nanopillar), and 35.07 for W (Hall device). The corresponding J_c_0 values are 80.65 MA cm^–2^, 33.88 MA cm^–2^, 70.71 MA cm^–2^, and 22.21 MA cm^–2^, respectively.

A comparative summary of various composite type-Z SOT systems that achieve field-free switching is presented in Table 1, including many key parameters. The competitive performance of the Co/W-based devices highlights the promise of this composite system as a highly efficient, thermally stable, and scalable platform readily adapted for large-area and CMOS-compatible fabrication.

**Table 1:** Benchmark of Field-Free Switching: Critical Dimension of Free Layer (nm), Switching Current Density (*J*_*c*_), Pulse Width (ms), External Symmetry-Breaking Field (Oe), Types of SOT, and FM Layers^22,29,30,41–43^

	CD (nm)	*J*_*c*_ (MA/cm^2^)	*t*_*pulse*_ (ms)	*H*_*x*_ (Oe)	SOT-type	FM (nm)	ref.
**Co/W4-8 (6 nm)**	**100**	**51**	**0.06**	**0**	**Z**	**CFB (1.2)**	**This work**
W(4)	860	20	50	1500	Z	CFB (1.4)	^42^
W(5)	120	136	100	200	Z	CFB (1.3)	^41^
W(3)/Pt(5)/W(1)	9000	30	1	3000	Z	CFB (1.1)	^29^
CFB(4)/Ti(3)	150	30	0.1	0	Z	CFB (1)	^43^
CFB(4)/Ti(3)	4000	26.6	0.015	0	Z	CFB (1)	^22^
W(2)/Ni_4_W(10)	10000	17	10	0	Z	Co (0.9)	^30^

This study establishes a compositional and dimensional design principle for realizing highly efficient field-free SOT switching in perpendicular magnetic systems. By tailoring the Co/W configuration within a fixed SOT-layer thickness, we achieve a field-free switching ratio exceeding 90%; second-harmonic Hall and AHE loop-shift measurements reveal unconventional torque components beyond the conventional in-plane spin polarization of W. First-principles calculations further show that Co/W dimensional modulation reconstructs the spin-resolved electronic structure, with an increased presence of W-projected out-of-plane *S*_*z*_^*W*^ character in the periodically modulated configuration, providing microscopic electronic-structure support for the experimentally observed modification of torque symmetry. Together with micromagnetic simulations, our results demonstrate that the field-free switching behavior is governed by the interplay between unconventional torque components and the magnetic-anisotropy configuration. Importantly, robust field-free switching is retained in a 100 nm Co/W nanopillar, demonstrating the scalability of this approach toward nanoscale SOT devices. These findings suggest that Co/W superlattice engineering provides an effective route for modifying torque symmetry and enabling field-free SOT switching, while offering a scalable materials platform for future SOT device integration.

## Supplementary Material



## Notes

The Supporting Information provides . The Supporting Information is available free of charge at https://pubs.acs.org/doi/10.1021/acs.nanolett.6c04408.

Details pertaining to current-induced magnetization switching in the W reference sample, multilayer stacking schemes of Co/W superlattice structures, field-dependent switching characteristics of Co/W SOT devices, switching phase diagrams for different Co repetitions, current-induced loop-shift measurements, first-principles calculations, second harmonic Hall measurements, macrospin and micromagnetic simulations, surface roughness characterization, anomalous Hall effect characterization of scaled W and Co/W SOT devices, and pulse-width-dependent switching behavior in scaled devices (PDF)
