# Supplementary material for: Dimensional Engineering of Co/W Superlattices Enables Out-of-Plane Spin Polarization with 90% Field-Free Switching in Perpendicular CoFeB
Source: Nano Lett. 2026 Oct 12;26(41):14482–90. doi: 10.1021/acs.nanolett.6c04408 (PMC13693498; doi:10.1021/acs.nanolett.6c04408)
Supplement: Supplementary file 1 [file nl6c04408_si_001.pdf]

Supporting Information

Dimensional Engineering of Co/W Superlattices Enables Out-of-Plane Spin Polarization with 90% Field-Free Switching in Perpendicular CoFeB

*Yu-Lon Lin[1], Jing-Wen Chen[1], Yan-Zhen Huang[1], Tzu-Chuan Hsin[2], Chih-Wei Cheng[1], Yu-Han Huang[3], Ssu-Yen Huang[4,5], Shintaro Hara[6], Tatsuya Kishi[6], and Yuan-Chieh Tseng[1]**

[1]Department of Materials Science & Engineering, National Yang-Ming Chiao-Tung University, Hsinchu 30010, Taiwan, ROC

[2]Department of Materials Science & Engineering, National Taiwan University, Taipei 10617, Taiwan, ROC

[3]Taiwan Semiconductor Research Institute, Hsinchu 30010, Taiwan, ROC

[4]Department of Physics, National Taiwan University, Taipei 10617, Taiwan, ROC

[5]Center of Atomic Initiatives for New Materials, National Taiwan University, Taipei 10617, Taiwan, ROC

[6]TopoLogic Inc., Tokyo 113-0033, Japan

S1 Current-induced magnetization switching of the W reference sample

Fig. S1(a) shows the AHE characterization of the W control sample; a clear hysteresis loop depicts a good PMA of the CFB. Fig. S1(b) presents the current-induced magnetization switching of the W control sample; an external symmetry-breaking field was applied to enable the magnetization reversal.

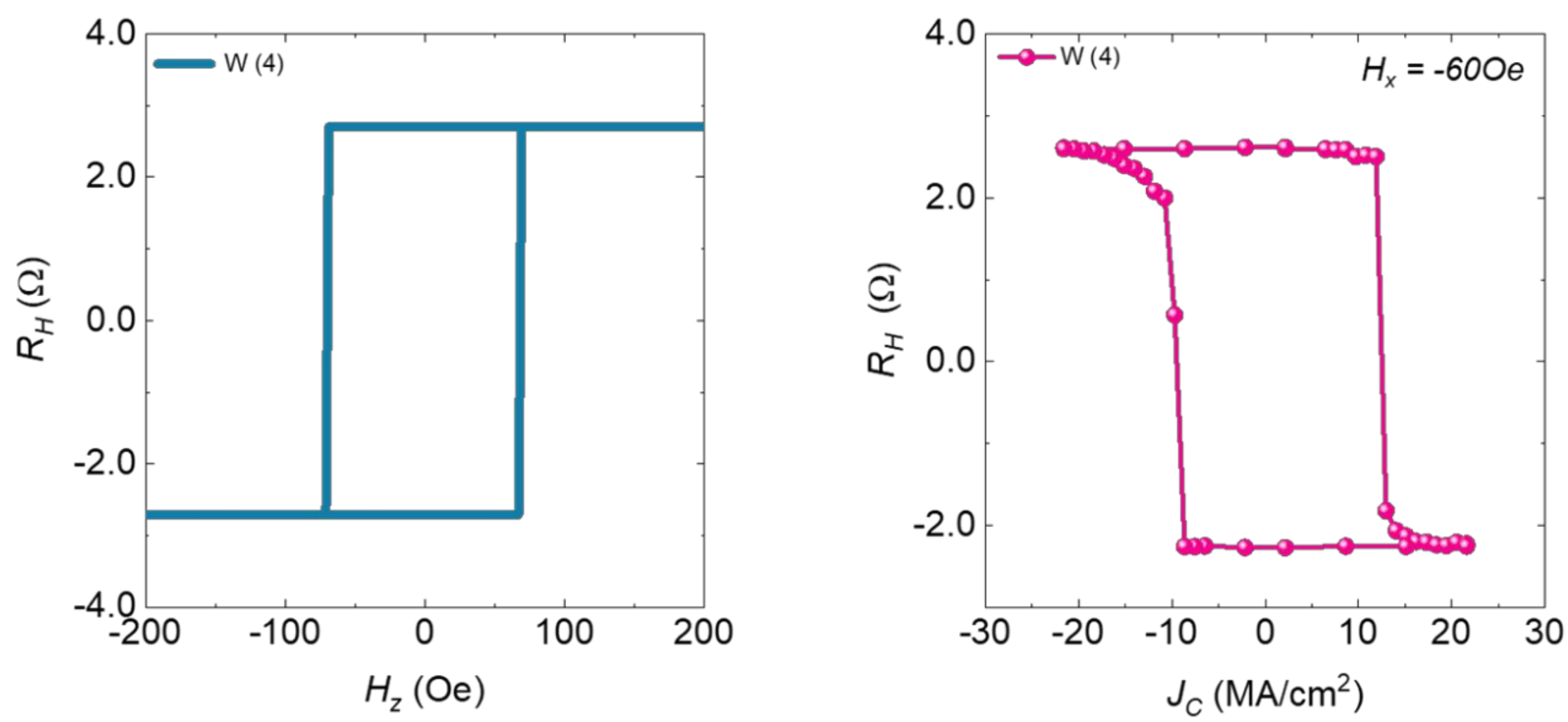

Fig. S1. Current-induced magnetization switching in W SOT devices. (a) AHE loop of control W(4nm) / CFB(1.2nm) SOT device. (b) Current-induced magnetization switching of the W SOT device under an external field of -60 Oe to assist the switching.

S2 Summary of Multilayer Stacking in Co/W Superlattice Structures

	W	Co/W 1-8	Co/W 2-8	Co/W 3-8	Co/W 4-8	Co/W 4-24
1	W (4)	W (2.77)	W (1.85)	W (1.39)	W (1.1)	W (0.96)
2		Co (0.45)	Co (0.23)	Co (0.15)	Co (0.11)	Co (0.3)
3		W (2.77)	W (1.85)	W (1.39)	W (1.1)	W (0.96)
4			Co (0.23)	Co (0.15)	Co (0.11)	Co (0.3)
5			W (1.85)	W (1.39)	W (1.1)	W (0.96)
6				Co (0.15)	Co (0.11)	Co (0.3)
7				W (1.39)	W (1.1)	W (0.96)
8					Co (0.11)	Co (0.3)
9					W (1.1)	W (0.96)

Fig. S2. Sequence of W and Co sublayer thicknesses in Co/W superlattice stacks. List of the alternating W and Co sublayer thicknesses employed in the superlattice growth of Co–W composite layers, along with the reference W sample. The numbers in parentheses denote the designed sublayer thickness for each W and Co layers in nanometers.

S3 Field-dependent current-induced magnetization switching in Co/W SOT Devices

Fig. S3(a)-(e) presents the current-induced magnetization switching behaviors of the fabricated Co/W SOT devices with external in-plane magnetic fields ranging from +200 Oe to −200 Oe. All devices exhibit a consistent switching chirality identical to that observed in conventional W-based SOT systems, confirming that the SOT polarity remains governed by the y-spin generated from the W component within the composite system.

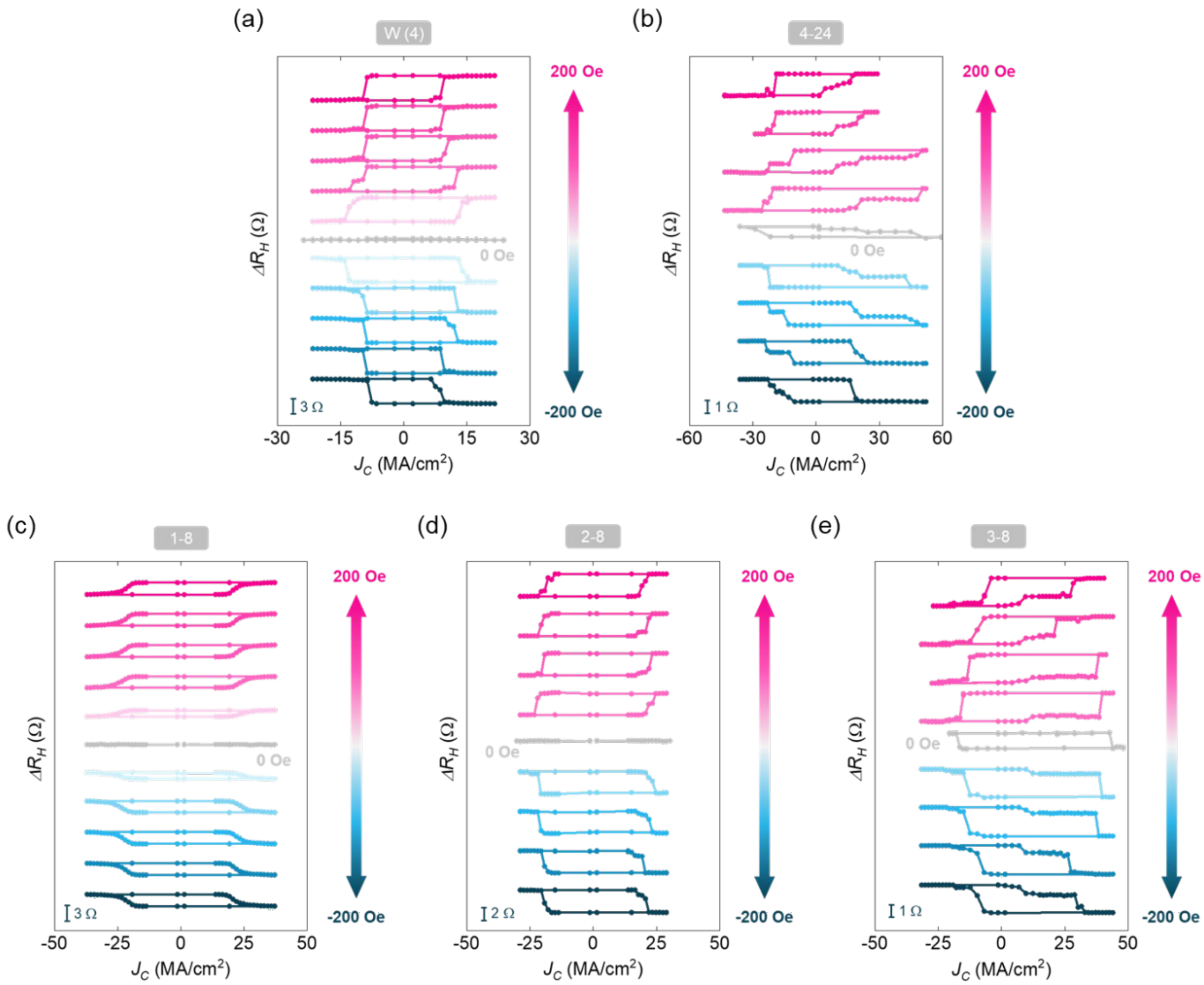

Fig. S3. Current-induced magnetization switching of W and Co/W SOT devices with external field ranging from +200 Oe to -200 Oe. (a) W (4nm), (b) Co/W4-24, (c) Co/W1-8, (d) Co/W2-8, and (e) Co/W3-8, respectively.

S4 Switching Phase Diagram (SPD) of various Co repetition in Co/W SOT Devices

Fig. S4 shows the SPD characteristics of Co/W SOT devices with the number of Co repetitions for two and three layers. Field-free switching was observed only in the three and four-layer repetitions of Co in the Co/W SOT device. Reducing the number of Co repetitions in a composite Co/W layer

appears to have conventional SOT behavior. This supports our conclusion that the SOT is mainly governed by the relatively thick adjacent W layer, which effectively masks the contribution from the z-polarized spin current generated by the low-dimensional Co sublayer.

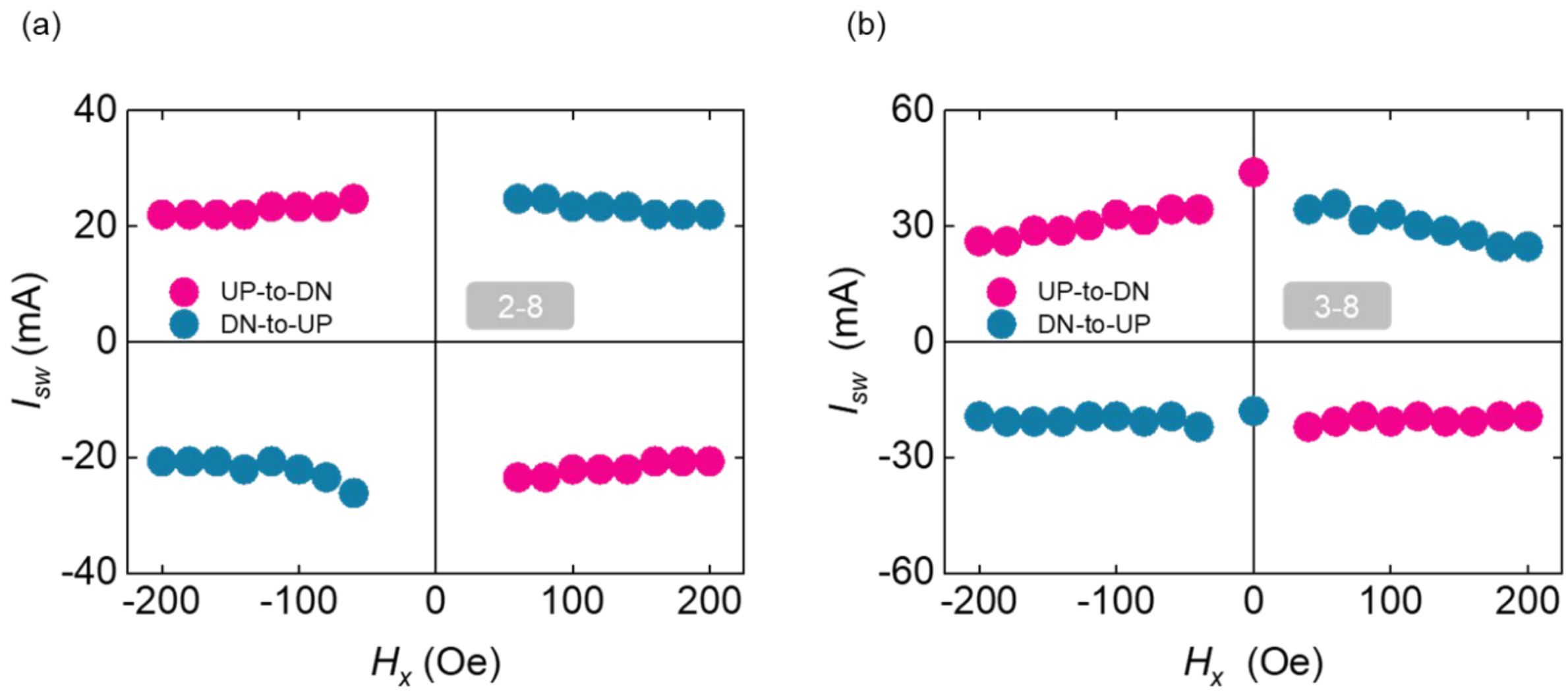

Fig. S4. SOT phase diagram of Co/W SOT devices with varying Co repetition in the SOT channel. Specifically, with (a) 2 and (b) 3 Co sublayers.

S5 Current-induced loop shift measurement of the Co/W SOT devices

Current-induced loop shift measurements were performed without applying an external in-plane field to confirm the generation of an out-of-plane spin current from the SOT layer. Fig. S5(a)-(e)

depicts the current-induced loop shift results of the fabricated W control sample and Co/W samples with varying the read current amplitude. In addition to the Co/W4-8 device discussed in the main text, effective perpendicular loop shifts were also observed in Co/W3-8 and Co/W4-24 samples, corroborating the presence of a z-oriented spin polarization. These results are consistent with the observed field-free switching behavior, highlighting the essential role of the low-dimensional Co sublayer in enabling such symmetry breaking.

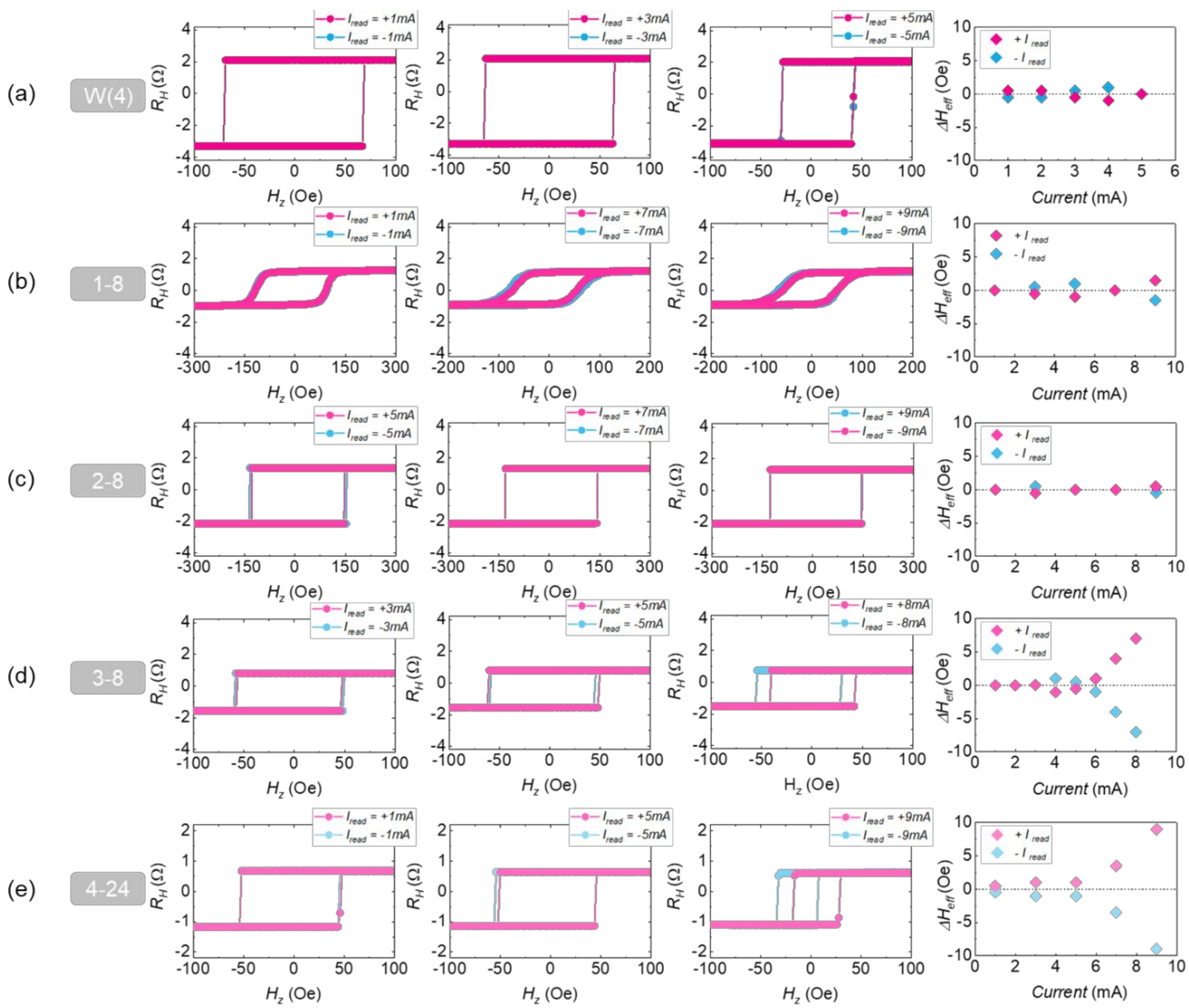

Fig. S5. Current-induced magnetic loop shift characterization of Co/W SOT devices. Current-induced loop-shift measurements performed under read current amplitudes ranging from 1 to 9 mA (1 to 5 mA for the W control sample). The corresponding perpendicular effective field (*H*eff)

was extracted as a function of the read current, summarizing the AHE loop-shift characteristics for (a) W (4 nm), (b) Co/W1-8, (c) Co/W2-8, (d) Co/W3-8, and (e) Co/W4-24.

S6 First-principles calculations of spin-resolved electronic structures in dimensionally modulated Co/W

First-principles calculations were performed within density functional theory (DFT) using the Vienna Ab initio Simulation Package (VASP) [1,2]. The exchange-correlation interaction was described using the Perdew–Burke–Ernzerhof generalized gradient approximation (GGA) [3], together with the projector augmented-wave (PAW) method [4,5]. A plane-wave cutoff energy of 450 eV and a Monkhorst–Pack [6] k-point mesh density of $2\pi \times 0.04$ Å^{-1} were employed. The structures were relaxed until the residual atomic forces were below 0.01 eV Å^{-1}, with an electronic convergence criterion of 1×10^{-5} eV. Spin-polarized calculations including spin–orbit coupling (SOC) were performed using the noncollinear formalism, with the Co magnetic moments oriented along the out-of-plane direction (z-axis). Spin-resolved band structures were subsequently obtained along the high-symmetry k-path and analyzed using VASPKIT [7].

We considered two Co/W model structures corresponding to the experimentally investigated Co/W1-8 and Co/W4-8 configurations. Configuration A, representing Co/W1-8, consists of a W/Co/W sandwich structure with a W:Co:W thickness ratio of approximately 25:4:25. Configuration B, representing Co/W4-8, consists of periodically alternating W and Co sublayers with a thickness ratio of approximately 10:1:10:1:10:1:10:1:10. The specified ratios refer to the relative layer thicknesses rather than the numbers of atomic layers. A sufficiently thick vacuum layer was introduced along the film growth direction (z-axis) to eliminate spurious periodic interactions between adjacent slabs. Spin-resolved electronic-structure analysis provides microscopic information on the momentum-dependent spin character of electronic states and has been employed to identify unconventional out-of-plane spin polarization in spin–orbit-coupled systems [8]. The band structures of Configurations A and B were calculated along their respective high-symmetry k-paths.

Figures S6-1 and S6-2 show the Co- and W-projected band structures with the spin characters resolved into the S_x, S_y, and S_z components for Configurations A and B, respectively. The transition from the single Co-sublayer configuration to the periodically modulated Co/W configuration produces a configuration-dependent reconstruction of the spin-resolved electronic states near the Fermi level. Finite W-projected S_z^W character is already present in Configuration A and becomes more pronounced in Configuration B. This evolution demonstrates that altering the Co/W dimensional periodicity enhances the out-of-plane spin character of the reconstructed W-derived electronic states. The calculated spin-projected band structures describe the equilibrium spin character of the electronic states and therefore should not be directly identified with the corresponding current-induced torque components.

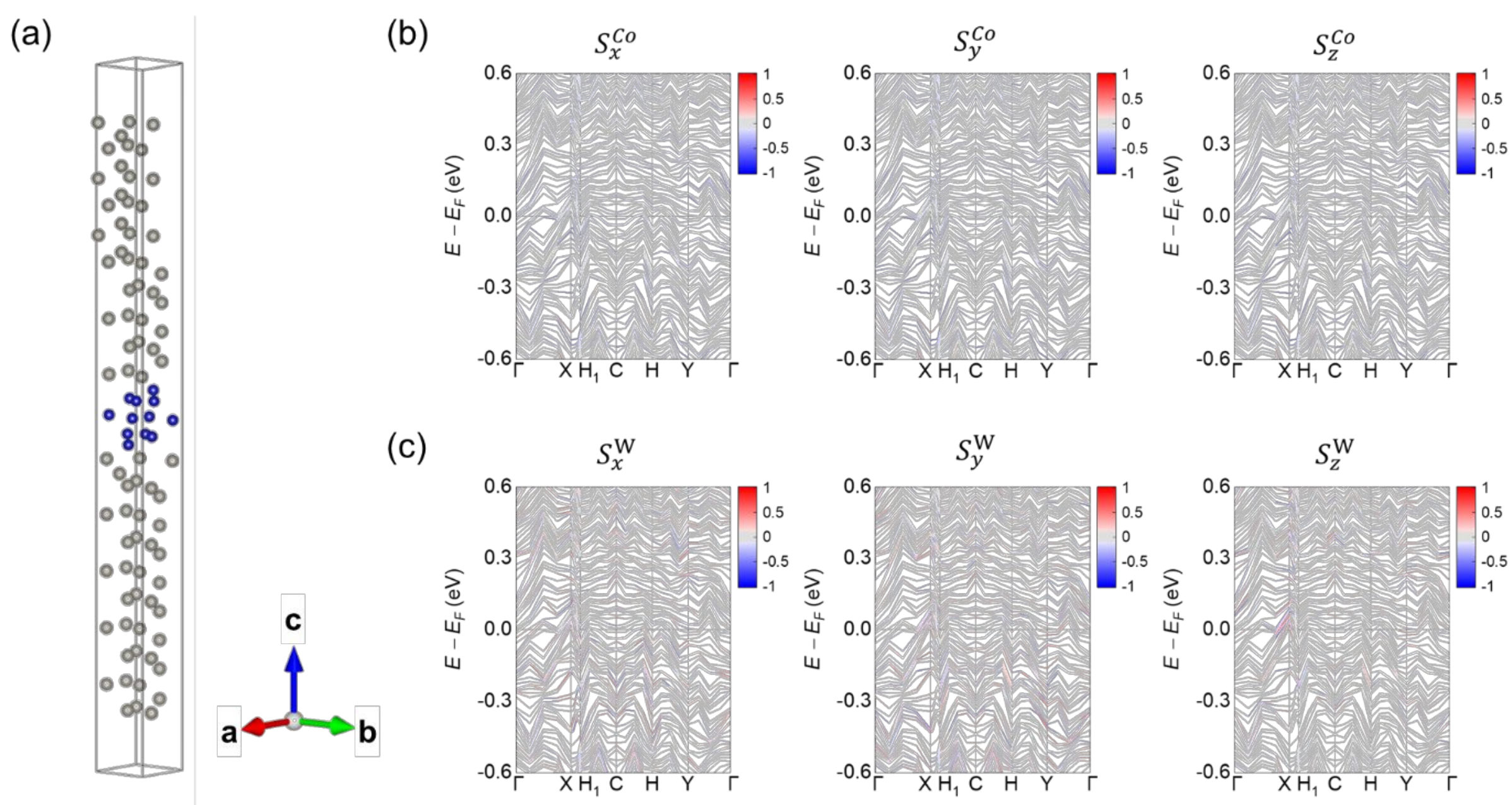

Fig. S6-1. Spin-resolved electronic structures of the Co/W Configuration A. (a) Atomic structure used in the first-principles calculation, where the gray and blue spheres represent W and Co atoms, respectively. (b) Co-projected (S_x^{Co}), (S_y^{Co}), and (S_z^{Co}) and (c) W-projected (S_x^{W}), (S_y^{W}), and (S_z^{W}) electronic band structures. The Fermi level is set to 0 eV. Red and blue indicate positive and negative spin projections, respectively.

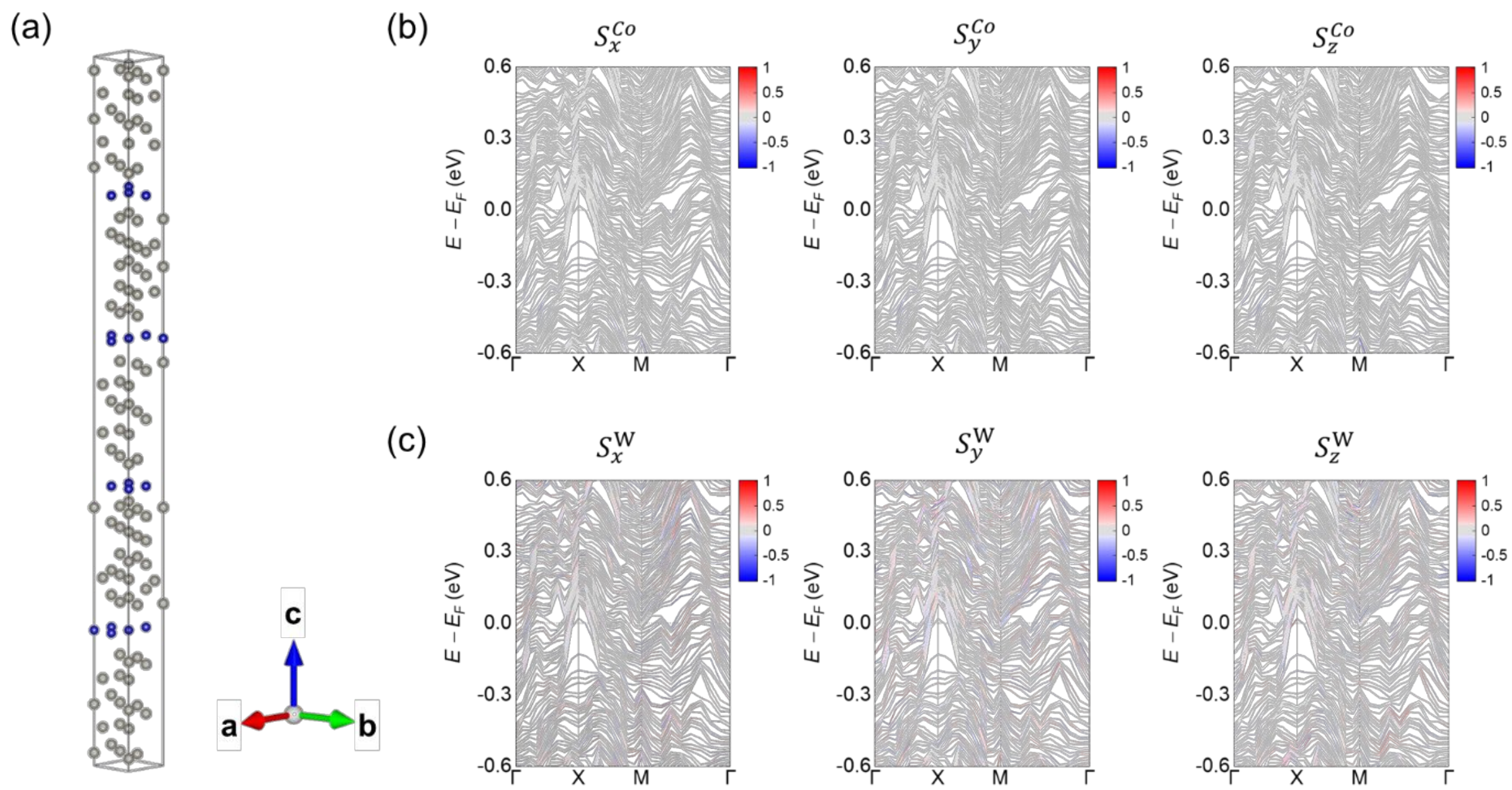

Fig. S6-2. Spin-resolved electronic structures of the Co/W Configuration B. (a) Atomic structure used in the first-principles calculation, where the gray and blue spheres represent W and Co atoms, respectively. (b) Co-projected (S_x^{Co}), (S_y^{Co}), and (S_z^{Co}) and (c) W-projected (S_x^{W}), (S_y^{W}), and (S_z^{W}) electronic band structures. The Fermi level is set to 0 eV. Red and blue indicate positive and negative spin projections, respectively.

S7 Quantification of SOT efficiencies by second-harmonic Hall analysis

Second-harmonic Hall measurements were performed on both Co/W and W samples using the differential planar Hall method to quantify the SOT effective fields [9-11]. The measurements were carried out on devices with a CoFeB thickness of 2.5 nm. An in-plane external field between 400 to 1800 Oe was applied at an angle of φ with respect to the current direction, as illustrated in Fig. S7-1(a). The first harmonic Hall response, which arises primarily from the planar Hall effect (PHE), is given by

$$R_{\omega} = R_{PHE} (1)$$

where $R_{PHE}$is the PHE amplitude. The second-harmonic Hall signal contains contributions from both damping-like and field-like torques, as well as thermoelectric effects, and can be expressed as [12-15]:

$$R_{2\omega} = R_{DL,y} cos\varphi + R_{FL,y} cos2\varphi cos\varphi + R_{DL,x} sin\varphi + R_{FL,x} cos2\varphi sin\varphi + R_{DL,z} cos2\varphi + R_{FL,z} (2)$$

where $R_{DL,x}$, $R_{DL,y}$, $R_{DL,z}$ are the second-harmonic response coefficients associated with damping-like torques generated by x, y, and z spins, respectively, and $R_{FL,x}$, $R_{FL,y}$, $R_{FL,z}$ are the field-like response coefficients.

Fig. S7-1(b)-(d) presents the AHE, first harmonic, and second harmonic responses of the Co/W4-8 device. While the conventional $R_{DL,y}$ and $R_{FL,y}$ terms can partially describe the angular dependence, a complete description requires additional damping-like components $R_{DL,x}$ and $R_{DL,z}$, providing clear evidence for unconventional spin polarization beyond the in-plane component. In contrast, the corresponding field-like terms $R_{FL,x}$ and $R_{FL,z}$ are negligible and do not contribute to the angular dependence.

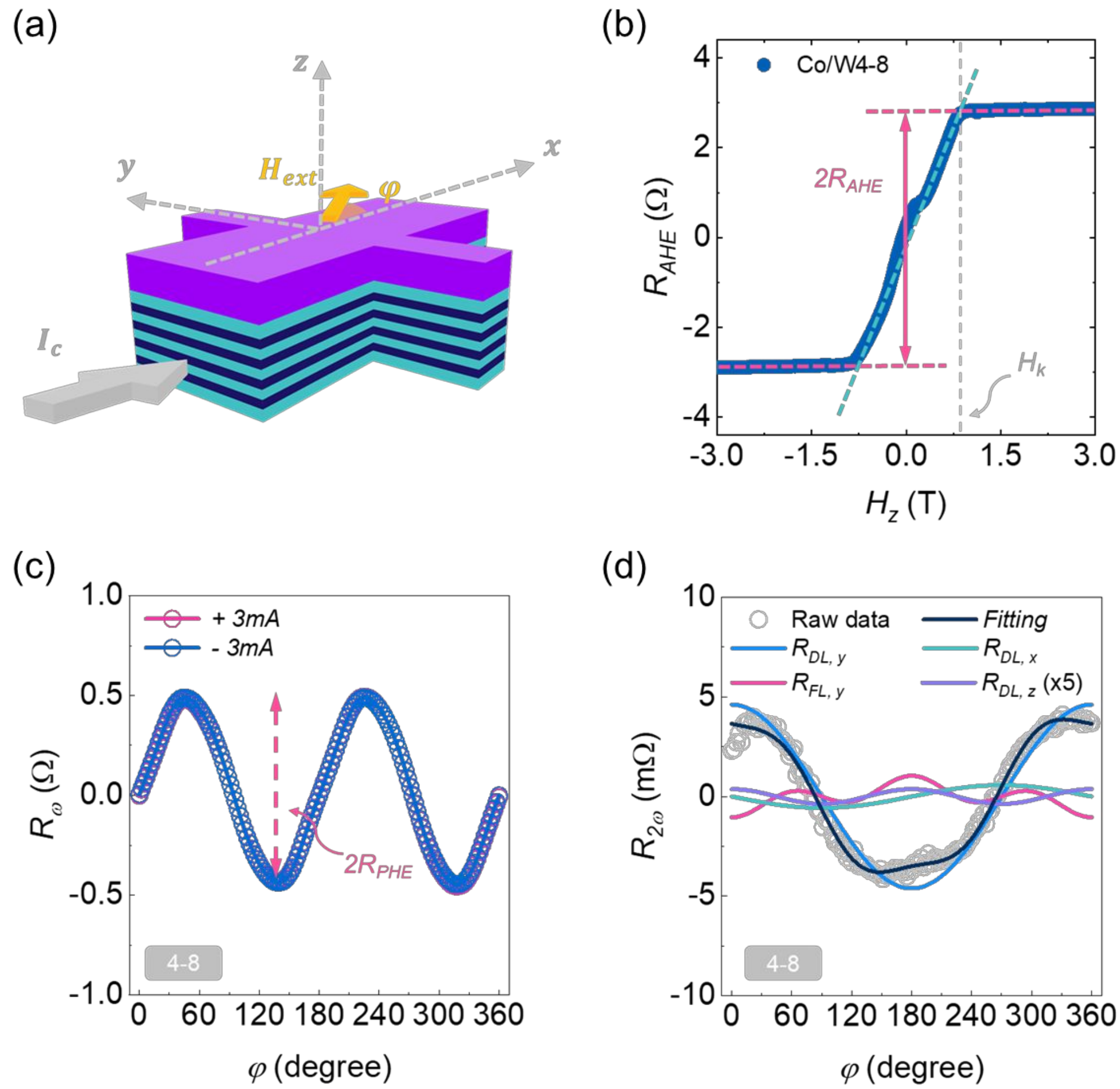

Fig. S7-1. Second-harmonic Hall analysis of Co/W4-8 SOT device. (a) Schematic illustration of the Co/W superlattice device and measurement configuration, where an in-plane magnetic field H_{ext} is applied at an angle ϕ with respect to the current direction. (b) Anomalous Hall resistance R_{AHE} as a function of out-of-plane magnetic field H_z, used to determine the magnetic anisotropy field H_k and the anomalous Hall resistance $2R_{AHE}$. (c) First-harmonic Hall resistance R_ω measured at $H_{ext} = 1600$ Oe as a function of ϕ under positive and negative current amplitude of 3mA, showing the expected sinusoidal $(sin2\varphi)$ angular dependence. (d) Second-harmonic Hall resistance $R_{2\omega}$ and corresponding fitting curves, where the contributions from $R_{DL,\,x}$, $R_{DL,\,y}$, $R_{DL,\,z}$, and $R_{FL,\,y}$ are extracted through angular decomposition.

Fig. S7-2(a)–(f) presents the first- and second- harmonic Hall responses of the pure W, Co/W1-8, and Co/W4-24 devices. A finite out-of-plane spin component is resolved in the optimized Co/W superlattice samples, whereas it is negligible in the W and Co/W1-8 reference samples (Fig. S7-2(d), (e)). No measurable out-of-plane contribution is observed in the W control and Co/W1-8 samples, confirming the absence of unconventional spin generation in these references.

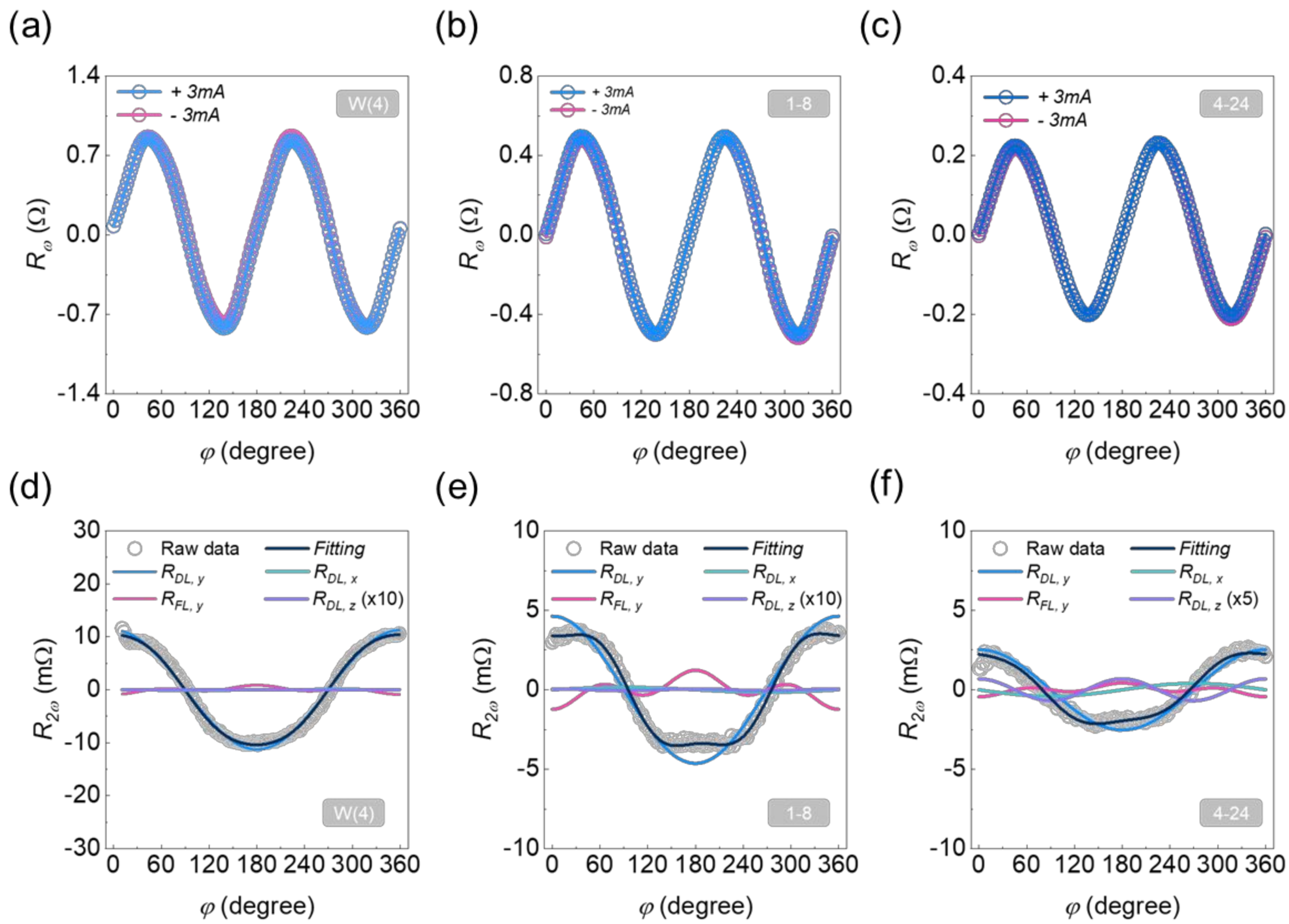

Fig. S7-2. First- and second-harmonic Hall responses of the pure W and Co/W superlattice devices under $H_{ext} = 1600$ Oe. (a)–(c) First-harmonic Hall signals for the pure W, Co/W1-8, and Co/W4-24 samples, respectively. (d)–(f) Corresponding second-harmonic Hall signals used for torque analysis.

To evaluate the SOT efficiencies along the x, y, and z directions, we carried out a field-dependent analysis of the harmonic Hall signals. The extracted resistance components exhibit linear scaling with the inverse magnetic field, enabling quantitative separation of the different torque contributions [11-14]:

$$R_{DL,y} = -\frac{\tau_{DL,0}^{y}}{\gamma}\frac{R_{AHE}}{H_{ext}+H_k} + R_{ANE} + R_{ONE}H_{ext} \quad (3)$$

$$R_{DL,x} = -\frac{\tau_{DL,0}^{x}}{\gamma}\frac{R_{AHE}}{H_{ext}+H_k} \quad (4)$$

$$R_{DL,z} = -\frac{\tau_{DL,0}^{z}}{\gamma}\frac{R_{PHE}}{H_{ext}} \quad (5)$$

$$R_{FL,y} = -R_{PHE}\frac{H_{FL,y}+H_{Oe}}{H_{ext}} \quad (6)$$

Here, $\tau_{DL,0}^{i}$ represents the effective-field amplitude associated with the i-polarized damping-like torque, where $i = x, y, z$, following previous multi-component second-harmonic Hall analyses[13,14]. Specifically, $\tau_{DL,0}^{x}$, $\tau_{DL,0}^{y}$, and $\tau_{DL,0}^{z}$ correspond to damping-like torque components generated by spin polarization along the x, y, and z directions, respectively, while $H_{FL,y}$ denotes the effective field associated with the field-like contribution from the y-polarized component. R_{AHE} and R_{PHE} are the anomalous and planar Hall resistances, respectively, while the H_k denotes the out-of-plane magnetic anisotropy field. R_{ANE} and R_{ONE} represent the anomalous Nernst and ordinary Nernst contribution, respectively. This analysis framework enables quantitative extraction of the multidirectional torque components, which are discussed below. The SOT efficiencies are defined as

$$\xi_{DL,i} = \frac{2e}{\hbar}\frac{\mu_0 M_s t_{FM}\tau_{DL,0}^{i}}{\gamma J_{SO}} \quad (7)$$

and

$$\xi_{FL} = \frac{2e}{\hbar}\frac{\mu_0 M_s t_{FM} H_{FL,y}}{J_{SO}} \quad (8)$$

where e is the elementary charge, $\hbar$ is the reduced Planck constant, μ_0 is the vacuum permeability, $M_s = 1100\ emu/c.c.$ is the saturation magnetization of CoFeB, t_{FM} is the thickness of the ferromagnetic layer, and J_{SO} is the charge current density in the spin-orbit layer. For the field-like component along y, the contribution from the Oersted field is subtracted. The Oersted field is estimated as

$$H_{Oe} = \frac{1}{2} J_{SO} t_{SO} (9)$$

where t_{SO} is the thickness of the SOT layer.

Beyond the conventional in-plane damping-like component $\xi_{DL,y}$, finite $\xi_{DL,x,}$ and $\xi_{DL,z}$ are resolved in the Co/W4-8 and Co/W4-24 superlattice devices. The corresponding damping-like SOT efficiencies are extracted from the field-dependent linear fitting of $R_{DL,x}$ and $R_{DL,z}$, as shown in Fig. S7-3. The $R_{DL,x}$ component exhibits a finite intercept in addition to the expected field-dependent contribution. This intercept may originate from thermal-related or other field-independent background contributions [16]. Since the SOT efficiency is determined from the field-dependent slope rather than the intercept, this offset does not affect the slope-based extraction of $\xi_{DL,x,}$. Quantitatively, $\xi_{DL,x}$ and $\xi_{DL,z}$ are -0.065 and -0.005 for Co/W4-8, and increase to -0.165 and -0.008 for Co/W4-24, respectively. The emergence of $\xi_{DL,z}$ provides direct evidence for an out-of-plane spin polarization, while the concurrent emergence of $\xi_{DL,x}$ indicates that the spin polarization deviates from the conventional in-plane σ_y direction. Such multi-axis damping-like torques are absent in the pure W and Co/W1-8 samples, but become pronounced in Co/W4-8 and Co/W4-24, highlighting the critical role of the superlattice structure. These results suggest that the spin polarization vector acquires a three-dimensional orientation due to symmetry breaking in the Co/W superlattice, leading to unconventional SOT behavior beyond the standard spin Hall framework.

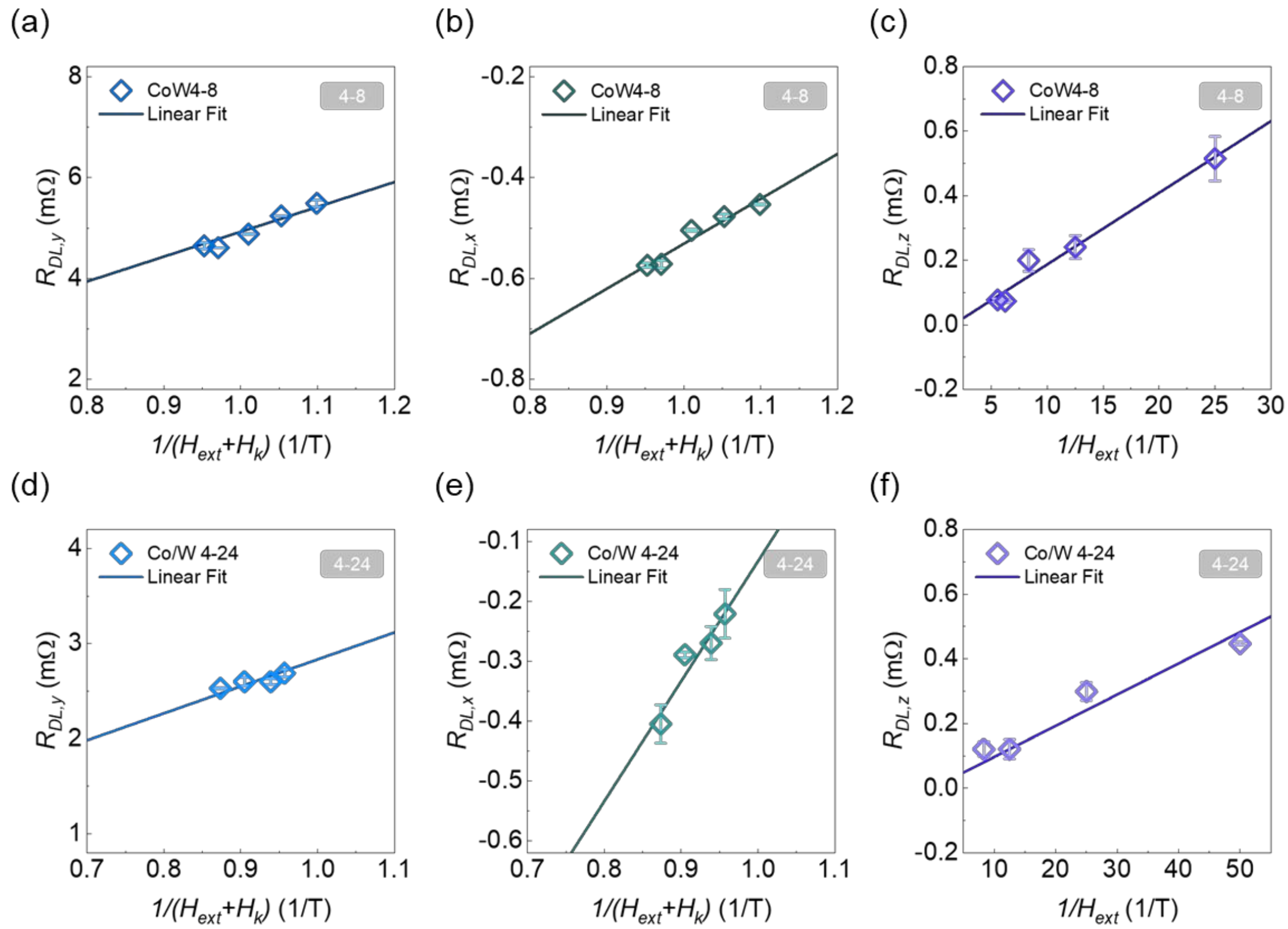

Fig. S7-3. Extracted damping-like SOT components along the x, y, and z directions for the (a)–(c) Co/W4-8 and (d)–(f) Co/W4-24 devices.

The damping-like and field-like SOT efficiencies along the y direction are extracted from the field-dependent linear fitting of $R_{DL,\,y}$ and $R_{FL,\,y}$, as shown in Fig. S7-4. The extracted $\xi_{DL,\,y}$ and $\xi_{FL,\,y}$ values are -0.378 and 0.038 for the pure W, -0.448 and 0.038 for Co/W1-8, -0.363 and 0.034 for Co/W4-8, and -0.238 and 0.040 for Co/W4-24, revealing a systematic evolution with the Co/W superlattice structure, as shown in Fig. S7-4. In contrast, the unconventional components $\xi_{DL,\,x}$ and $\xi_{DL,\,z}$ are negligible in pure W and Co/W1-8 but become finite in Co/W4-8 and Co/W4-24, indicating that the spin-current symmetry evolves with Co/W dimensional modulation. The experimentally resolved out-of-plane $\xi_{DL,\,z}$ component in Co/W4-8 is consistent with the increased W-projected S_z^W character revealed by the first-principles calculations, further supporting the role

of Co/W dimensional modulation in modifying the spin-resolved electronic structure and torque symmetry. Although Co/W4-24 exhibits a larger unconventional component, Co/W4-8 provides the best balance between conventional damping-like torque, perpendicular magnetic properties, and switching performance. The field-like response shows a small finite intercept in addition to the expected $1/H_{ext}$ dependence. Such behavior is commonly observed in harmonic Hall measurements and is attributed to weak field-independent contributions. As these contributions do not scale with $1/H_{ext}$, they do not affect the extraction of $\xi_{FL,y}$, which is determined from the slope of the linear fit. The extracted values are summarized in Table S1.

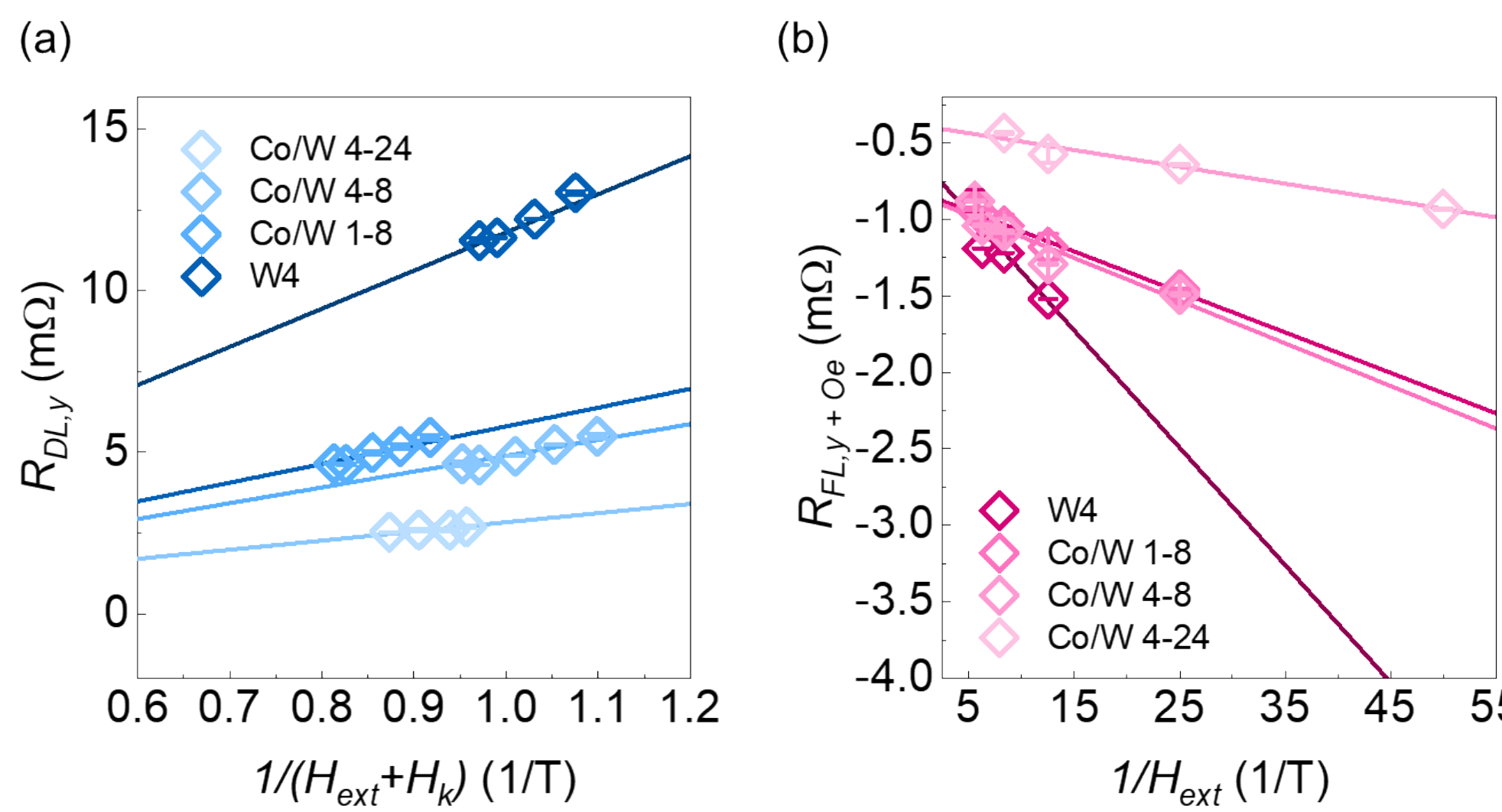

Fig. S7-4. Extracted (a) DL, and (b) FL efficiency from second harmonic Hall analysis for W(4), Co/W1-8, Co/W4-8, and Co/W4-24 samples.

Table S1. Summary table for all the terms extracted from the second harmonic Hall analysis.

	H_k (Oe)	R_{AHE} (Ω)	R_{PHE} (Ω)	$\xi_{DL,y}$	$\xi_{FL,y}$	$\xi_{DL,x}$	$\xi_{DL,z}$

W4	8500	5.4	0.83	-0.378	0.038	~ 0	~ 0
Co/W 1-8	10500	2.7	0.5	-0.448	0.038	~ 0	~ 0
Co/W 4-8	8700	2.85	0.88	-0.363	0.034	-0.065	-0.005
Co/W 4-24	10250	2.5	0.2	-0.238	0.04	-0.165	-0.008

S8 Macrospin and micromagnetic simulations

We first employed a macrospin model to simulate the spin dynamics in a T-shaped structure, defined as a hybrid structure in which a perpendicularly magnetized (PMA) layer is coupled to an in-plane magnetic anisotropy (IMA) layer, focusing on the easy-axis deflection arising from the coupling between the PMA layer and the IMA layer.

We employ a macrospin model based on the Landau–Lifshitz–Gilbert (LLG) equation to analyze the magnetization dynamics and the influence of the spin Hall effect. The LLG equation is written as:

$$\frac{\partial \vec{m}}{\partial t} = -\left(\vec{m} \times \vec{H}_{eff}\right) + \alpha \left(\vec{m} \times \frac{\vec{m}}{t}\right)(10)$$

By using the cross product of m on both sides, the above expression can be rearranged into the following form:

$$\frac{\partial \vec{m}}{\partial t} = -\frac{1}{1+\alpha^2}\left(\vec{m} \times \vec{H}_{eff}\right) - \frac{\alpha}{1+\alpha^2}\left(\vec{m} \times \left(\vec{m} \times \vec{H}_{eff}\right)\right)(11)$$

The effective field is given by

$$\vec{H}_{eff} = \gamma M_s\left[H_K\left(\vec{m} \cdot \hat{e_z}\right)\hat{e_z} + \vec{H}_x - \tau_{DL}\left(\vec{m} \times \left(\vec{z} \times \hat{j}\right)\right)\right],$$

where the three terms correspond to perpendicular magnetic anisotropy, an in-plane external field, and the damping-like spin–orbit torque (SOT), respectively. The SOT effective field is

$$\tau_{DL} = \frac{\hbar\theta_{DL}}{2e\mathrm{Ms}t_F}(12)$$

Here, $\vec{m} = \frac{dM}{dM_s}$ is the unit vector along the direction of magnetization, M is mthe agnetic moment, Ms is the saturated magnetization value, where γ is the gyromagnetic ratio, α stands for the Gilbert damping constant, ħ represents reduced Planck constant, e represents the electron charge, μ_0 is the permeability of vacuum, and t is the effective thickness of the free layer. To more accurately represent real-world situations, we utilize the parameters listed in Table I.

Using the condition $\vec{m} \times \vec{H}_{eff} = 0$, we obtained the real and analytical solutions of Eq. (2), which allowed us to determine the expression for the switching current density,

$$J_{sw} = \frac{2e\mathrm{Ms}t_F}{\hbar\theta_{DL}}\sqrt{\frac{\mathrm{H_K}^2}{32}\left[8 + 20(\frac{\mathrm{H_x}}{\mathrm{H_K}})^2 - (\frac{\mathrm{H_x}}{\mathrm{H_K}})^4 - (\frac{\mathrm{H_x}}{\mathrm{H_K}})(8 + (\frac{\mathrm{H_x}}{\mathrm{H_K}})^2)^{\frac{3}{2}}\right]}(13)$$

When $\mathrm{H_x} \ll \mathrm{H_K}$,

$$J_{sw} = \frac{2e\mathrm{Ms}t_F}{\hbar\theta_{DL}}\left(\frac{\mathrm{H_K}}{2} - \frac{H_x}{\sqrt{2}}\right)(14)$$

In the parameter settings of the macroscopic spin model, $\mathrm{H_K}$ is configured to 100 Oe. When the easy magnetization axis is rotated by φ degrees along the positive (or negative) x-axis, $H_K(\varphi) = H_K[(m_x,m_y,m_z)\cdot(\sin(\varphi),0,\cos(\varphi))](\sin(\varphi),0,\cos(\varphi))$.

Table S2. Detailed parameters adopted in the macrospin model.

	Symbol (unit)	Macrospin simulation
Saturation magnetization	M_s (emu/cc)	1000
Anisotropy field	H_K (Oe)	100
Damping constant	α	0.1
DLT efficiency	ξ_{DL}	0.25
FM thickness	t_{FM} (nm)	1
Switching current density	J_c (A/m^2)	$\pm 2 \times 10^{11}$
Vacuum permeability	μ_0 (H/m)	1.256×10^{-6}
Gyromagnetic ratio	γ $(T^{-1}s^{-1})$	1.76×10^{11}
Electron charge	e (C)	1.602×10^{-19}

In the micromagnetic simulation, an electric current was applied along the easy axis of the IMA layer (+x direction). As shown in Fig. S8(a), the calculated results reveal that the deflection of the PMA easy axis leads to a pronounced shift in its current-induced magnetization reversal loop. This behavior is consistent with our experimental observations for samples containing 24% Co, implying that increasing the Co content in the Co/W underlayer introduces an in-plane magnetic component that tilts the easy axis of the upper PMA layer. When the magnetic easy axis lies along the in-plane x direction, magnetization reversal is governed by the combined action of an external field applied along x and the spin Hall effect, which generates an effective field along the easy axis to induce switching via damping-like torque precession. Even when the easy axis tilts from the out-of-plane z direction toward x, forming a canted PMA configuration, the underlying switching mechanism remains analogous to that in purely perpendicular or in-plane systems.

Therefore, an external magnetic field remains necessary to achieve deterministic SOT switching in such tilted states.

To further explore this effect, we examined how positive and negative deflection angles ($\pm\varphi$) of the easy axis influence the system, as shown in Fig. S8(b). The simulation results show that reversing the sign of φ results in a mirrored flip of the magnetization reversal curve, effectively reversing the associated phase diagram. Comparison with the experimental SPD of the Co/W4-24 sample shows that the observed current-direction asymmetry is more consistent with the negative-φ configuration in the simulation, corresponding to an easy-axis canting toward the −x direction under the present coordinate convention. This may be due to the initial magnetization orientation chosen in the IMA layer. A comparative analysis of the switching phase diagrams reveals that tilting the easy axis shifts the critical switching current toward the negative direction; however, the overall symmetry of the diagram is preserved.

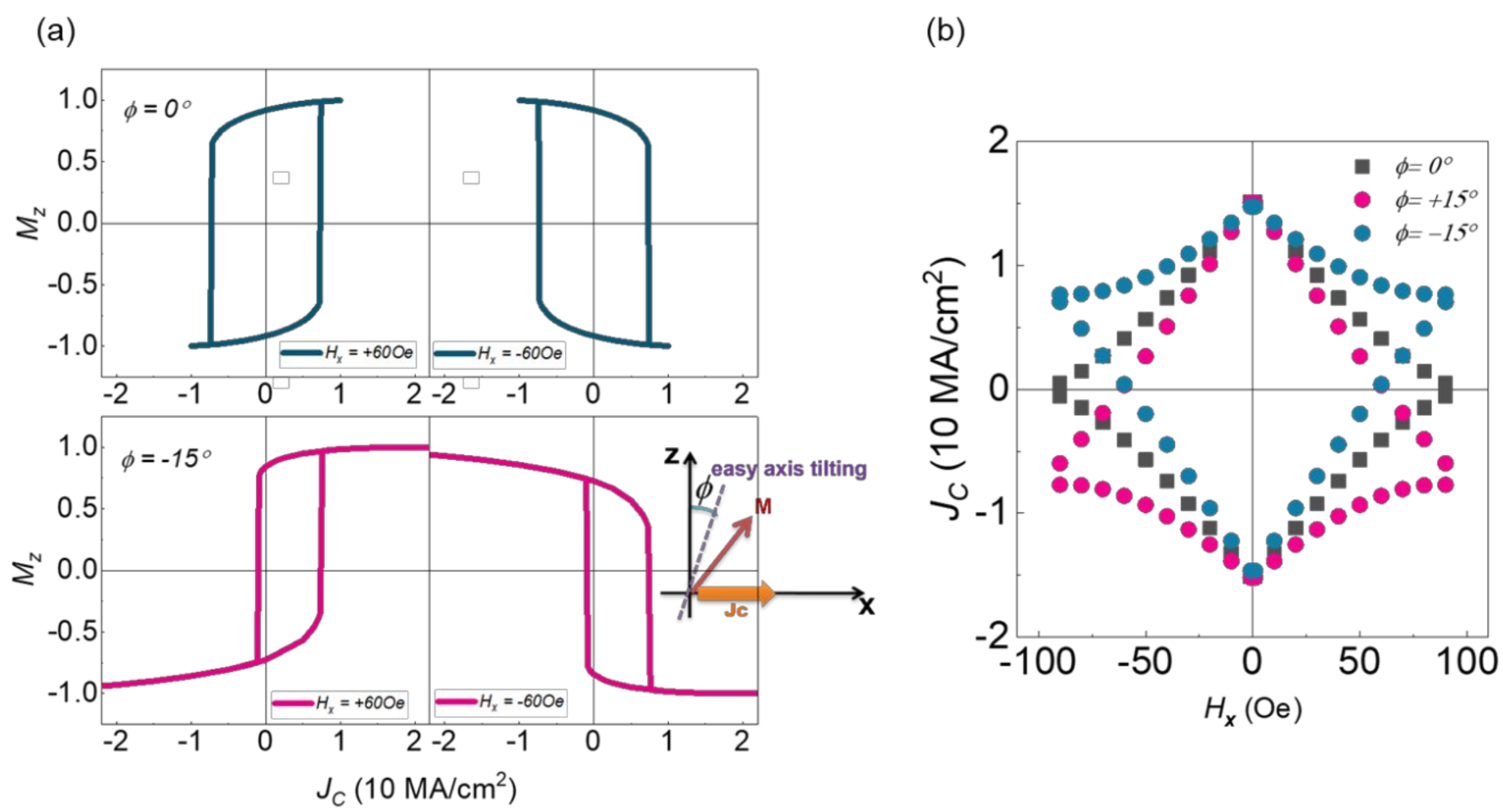

Fig. S8. Simulation of current-induced magnetization switching. (a) Simulated switching characteristics under two representative conditions. The upper panel shows the conventional PMA SOT switching, whereas the lower panel illustrates the type-T mode, in which the PMA easy axis

is deflected due to IMA coupling. The inset indicates the applied current direction (+x) and the corresponding tilt of the PMA easy axis. (b) Simulated switching phase diagrams (SPDs) showing the variation of the critical current density J_{sw} as a function of the in-plane magnetic field component H_x for different easy-axis deflection angles (ϕ).

To quantitatively examine the interaction between z-spin polarization and PMA tilting under zero external magnetic field, Micromagnetic simulations were performed using OOMMF with the Xf_SpinXferEvolve extension to investigate SOT–driven switching in ferromagnetic/half-metallic (FM/HM) heterostructures. The model consists of a perpendicularly magnetized FM thin film (280 × 140 nm²). Material parameters, including the saturation magnetization M_s, exchange stiffness A_{ex}, perpendicular anisotropy $K_{u,FM}$, and the damping-like spin Hall angle ξ_{DL}, are summarized in Table II. Magnetization dynamics are governed by the Landau–Lifshitz–Gilbert equation augmented by damping-like and field-like SOT terms (Eq. 6).

$$\frac{dm}{dt} = -\gamma m \times H_{eff} + \alpha m \times \frac{dm}{dt} + \frac{\gamma \hbar J_c}{2e\mu_0 M_s t_F}[\xi_{DL} m \times (\sigma \times \xi_{DL} m) + \xi_{FL} \sigma \times m] \quad (15)$$

Where t_{FM} represents the thickness of the ferromagnetic layer, m is the unit vector indicating magnetization, ξ_{DL} is the effective spin Hall angle dependent on the material, and σ is the related spin polarization.

In a typical NM/FM system with a heavy-metal spin-source layer, the charge current flows along the x-axis. As a result, the spin current flows along the z-axis, and the spin moment flows along the y-axis. Due to the symmetry constraints of conventional heavy metals, the charge current can only generate spin currents with spin moments σ_y oriented in the y-direction. Therefore, there are no spin moments along the x- or z-directions. However, if the current-induced spin current has

some of its spin moment along the z-direction, it can produce unconventional spin-orbit moments that can significantly enhance the efficiency of the PMA magnetized switch. In this simulation, we examined how the spin polarization angle and the tilt of the easy magnetization axis influence flipping efficiency. The spin polarization direction is given by σ = (0, cosη, sinη), where η represents the spin polarization angle affecting the strength of the z-spin component. The easy magnetization axis tilt is characterized by tilting angles $(\sin\varphi, 0, \cos\varphi)$, and the outcomes are shown in a phase diagram.

Table S3. Detailed parameters adopted in micromagnetic simulation.

	Symbol (unit)	Micromagnetic simulation
Saturation magnetization	$M_s\ (A/m)$	1×10^6
Uniaxial-anisotropy energy density	$K_U\ (J/m^3)$	8.2×10^5
Damping constant	α	0.1
Exchange-stiffness constant	$A_{ex}\ (J/m)$	2×10^{-11}
DLT efficiency	ξ_{DL}	0.3

FM size $(L \times W \times H)$	$V_{cuboid}\ (nm^3)$	$(280 \times 140 \times 1)$
Mesh size $(L \times W \times H)$	$V_{cell}\ (nm^3)$	$(2 \times 2 \times 1)$

S9 Roughness Characterization of Co/W SOT Devices

Fig. S9 presents the AFM characterization of W control sample with and the Co/W 4-8 sample, where the surface roughness (R_q) was determined to be 0.240 and 0.207, respectively.

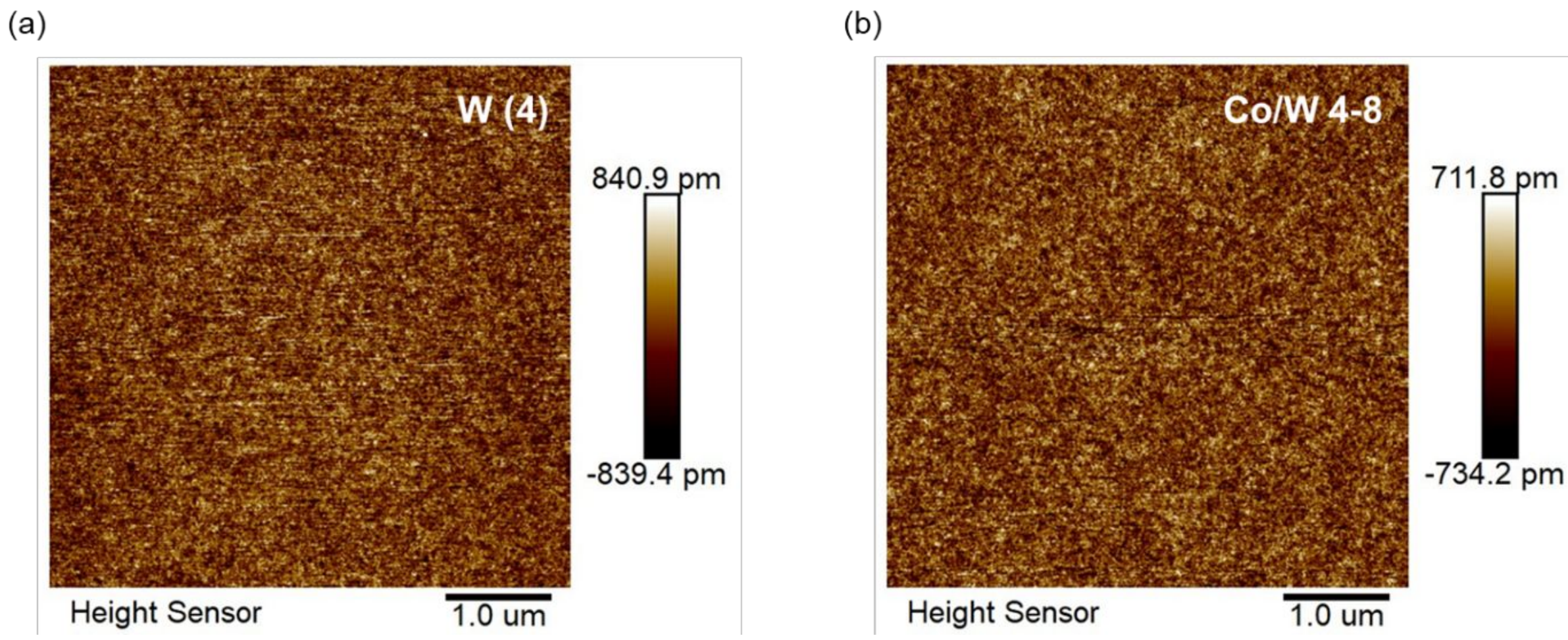

Fig. S9. Atomic force microscopy (AFM) characterization of Co/W films. Representative AFM topographic images of W and Co/W films of **a.** W (4) **b.** Co/W4-8 samples, acquired over a 5×5 μm^2 scan area.

S10 AHE characterization of the scaled W and Co/W SOT devices

Fig. S10 presents the AHE characteristics of W and Co/W4-8 SOT devices, fabricated in both Hall-bar (10 μm) and nanopillar (100 nm) geometries. The anomalous Hall resistance was recorded

under a DC bias of 200 μA. A significant enhancement of magnetic coercivity is observed in the nanopillar (100 nm) devices for both W and Co/W4-8 systems.

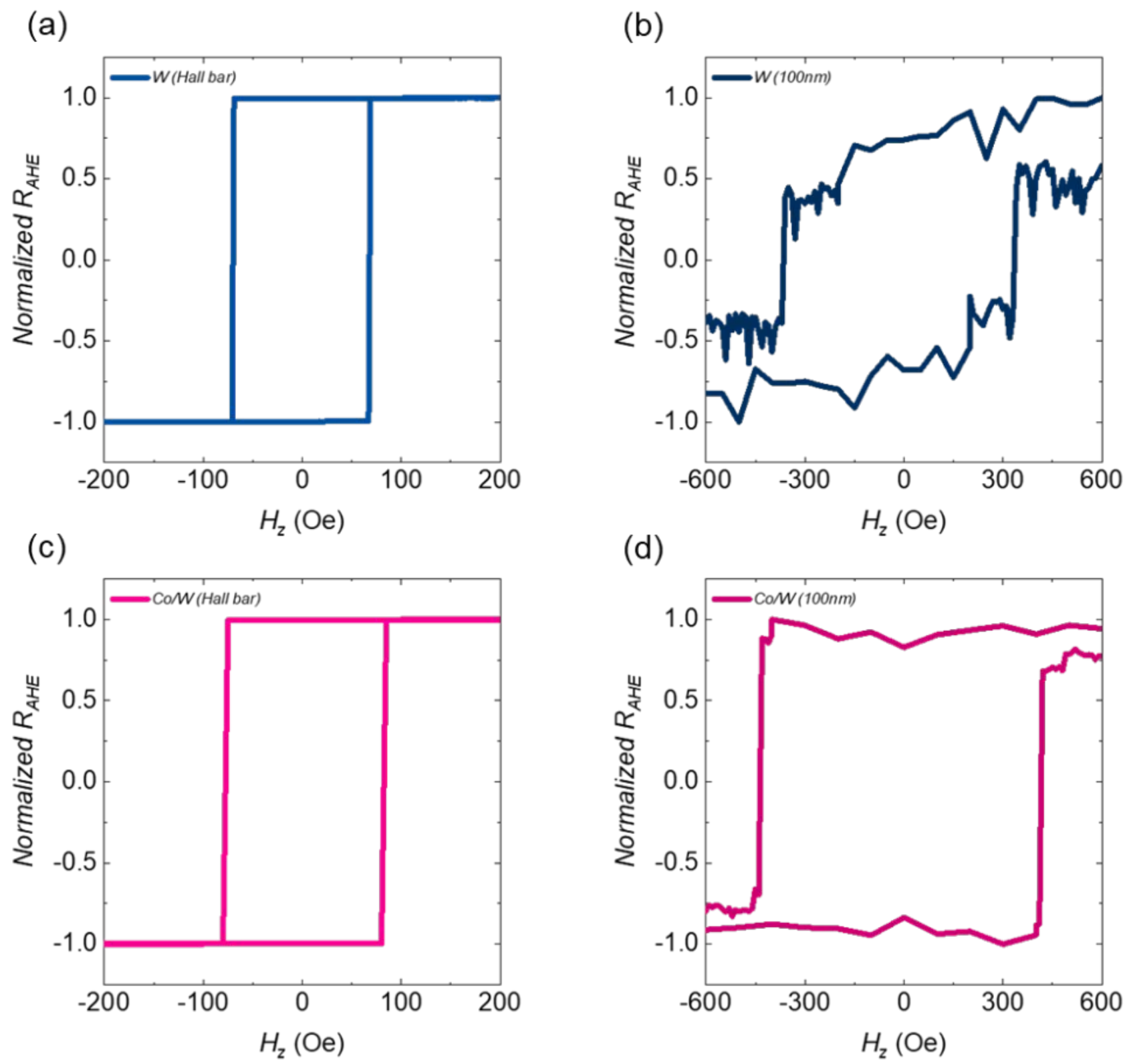

Fig. S10. AHE characterization of scaled SOT devices. Hall resistance as a function of external magnetic field along the z-axis of (a)(b) W and (c)(d) Co/W4-8 SOT devices in which the nanopillar dimensions were scaled from 10 μm (Hall bar) to 100 nm (nanopillar).

S11 Pulse-width dependence of current-induced magnetization switching in scaled devices

Current-induced magnetization switching was examined in both W and Co/W4-8 SOT devices, fabricated in Hall-bar (10 μm) and nanopillar (100 nm) geometries. The write current pulse width was systematically varied from 60 μs to 1 ms to probe the switching dynamics (see Fig. S11).

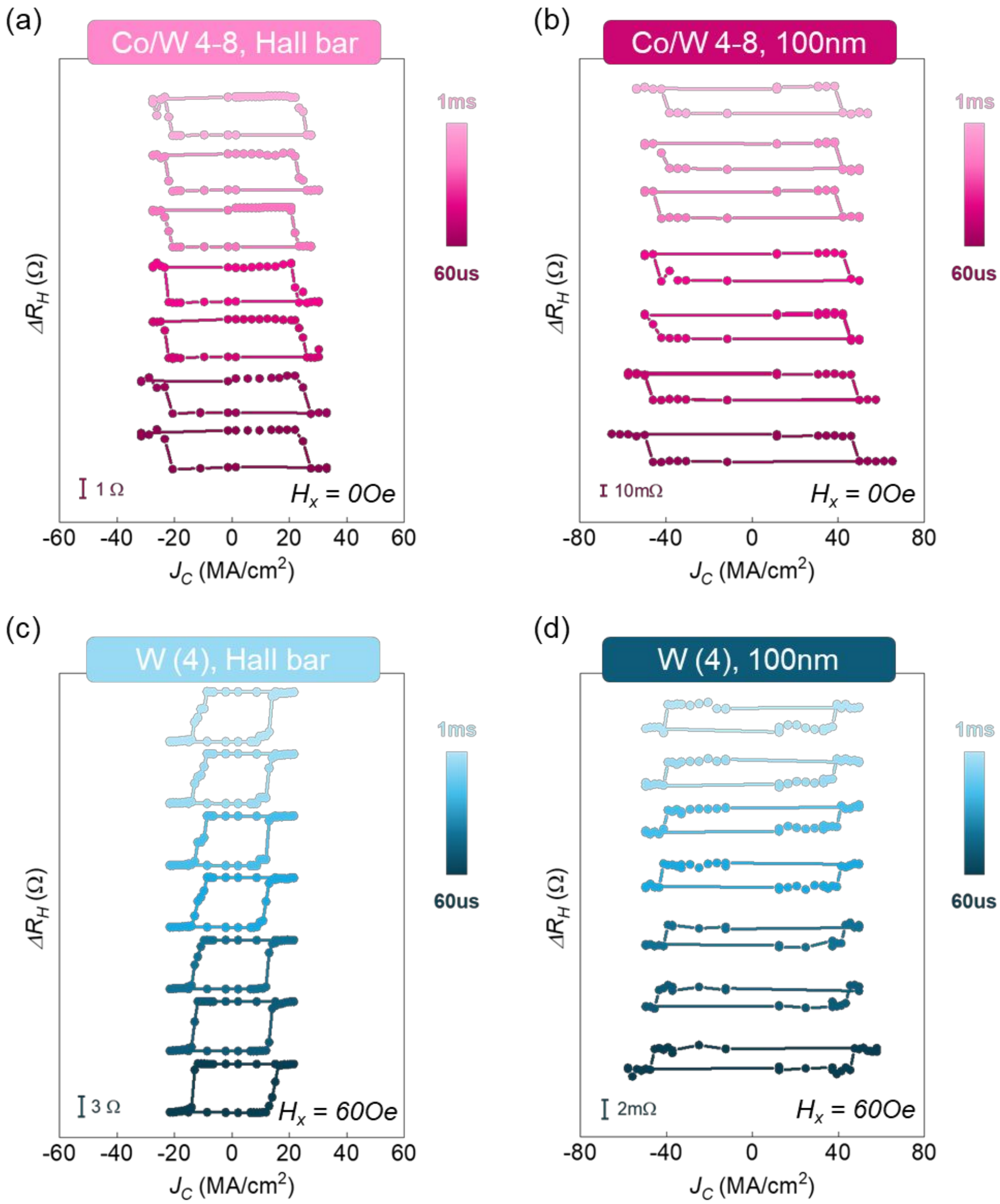

Fig. S11. Current-induced SOT switching in scaled W and Co/W SOT devices. Representative current-induced magnetization switching characteristics for Hall bar and nanopillar devices

employing (a)(b) W and (c)(d) Co/W4-8 SOT channels, respectively. For the W-based SOT device, an external magnetic field of -60 Oe was applied to assist the magnetization switching.

REFERENCES

(1) Kresse, G.; Furthmüller, J. Efficient iterative schemes for ab initio total-energy calculations using a plane-wave basis set. *Phys. Rev. B* **1996**, *54*(16), 11169–11186.

(2) Kresse, G.; Hafner, J. *Ab initio* molecular dynamics for liquid metals. *Phys. Rev. B* **1993**, *47*(1), 558–561.

(3) Perdew, J. P.; Burke, K.; Ernzerhof, M. Generalized Gradient Approximation Made Simple. *Phys. Rev. Lett.* **1996**, *77*(18), 3865–3868.

(4) Blöchl, P. E. From ultrasoft pseudopotentials to the projector augmented-wave method. *Phys. Rev. B* **1994**, *50*(24), 17953–17979.

(5) Kresse, G.; Joubert, D. From ultrasoft pseudopotentials to the projector augmented-wave method. *Phys. Rev. B* **1999**, *59*(3), 1758–1775.

(6) Monkhorst, H. J.; Pack, J. D. Special points for Brillouin-zone integrations. *Phys. Rev. B* **1976**, *13*(12), 5188–5192.

(7) Wang, V.; Xu, N.; Liu, J.-C.; Tang, G.; Geng, W.-T. VASPKIT: A user-friendly interface facilitating high-throughput computing and analysis using VASP code. *Comput. Phys. Commun.* **2021,** 267, 108033

(8) Cao, P.; Xie, S.-Y.; Li, X.-B.; Jiang, Y. Pure Out-of-Plane Spin Polarization Induced by Rashba-Type Splitting. *Nano Lett.* **2025**, *25*(26), 10287–10293.

(9) DC, M.; Grassi, R.; Chen, Y. J.; Jamali, M.; Hickey, D. R.; Zhang, D.; Zhao, Z.; Li, H.; Quarterman, P.; Lv, Y.; Li, M.; Manchon, A.; Mkhoyan, K. A.; Wang, J.-P. Room-temperature high spin–orbit torque due to quantum confinement in sputtered $Bi_xSe_{(1-x)}$ films. *Nat. Mater.* **2018**, *17*(9), 800–807.

(10) Ojha, D. K.; Chatterjee, R.; Lin, Y.-L.; Wu, Y.-H.; Chen, P.-W.; Tseng, Y.-C. Spin-torque efficiency enhanced in sputtered topological insulator by interface engineering. *J. Mag. Magn. Mater.* **2023**, *572*, 170638.

(11) Chang, H.-K.; Chi, K.-Y.; Lin, Y.-L.; Lai, Y.-H.; Huang, Y.-L.; Pai, C.-F.; Yang, C.-Y. Spin–Orbit torque booster in an antiferromagnet via facilitating a global antiferromagnetic order: A route toward an Energy-Efficient memory. *ACS Appl. Mater. Interfaces* **2024**, *16*(47), 65037–65045.

(12) Avci, C. O.; Garello, K.; Gabureac, M.; Ghosh, A.; Fuhrer, A.; Alvarado, S.; Gambardella, P. Interplay of spin-orbit torque and thermoelectric effects in ferromagnet/normal-metal bilayers. *Phys. Rev. B* **2014**, *90*(22), 224427.

(13) Hayashi, M.; Kim, J.; Yamanouchi, M.; Ohno, H. Quantitative characterization of the spin-orbit torque using harmonic Hall voltage measurements. *Phys. Rev. B* **2014**, *89*(14), 144425.

(14) Bose, A.; Schreiber N. J.; Jain, R.; Shao, D. F.; Nair, H. P.; Sun, J.; Zhang, X. S.; Muller, D. A.; Tsymbal, E. Y.; Schlom, D. G.; Ralph, D. C. Tilted spin current generated by the collinear antiferromagnet ruthenium dioxide. *Nat. Electron.* **2022**, *5*(5), 267-274.

(15) Lu, S.; Zhu, M.; Wu, C.; Meng, D.; Gu, N.; Xiang, L.; Su, Y.; Zhang, J.; Liu, Y.; Xiong, R.; Feng, J.; Wu, H.; Chen, S.; Zhang, J.; Liang, S. Enhancement of the Unconventional Spin−Orbit Torque Efficiency through Kagome Orientation Engineering in Epitaxial Noncollinear Antiferromagnet Mn_3Sn. *Nano Lett.* **2026**, *26*(4), 1220-1227.

(16) Yang, Y.; Lee, S.; Chen, Y. C.; Jia, Q.; Dixit, B.; Sousa, D.; Odlyzko, M.; Garcia-Barriocanal, J.; Yu, G.; Haugstad, G.; Fan, Y.; Huang, Y.-H.; Lyu, D.; Cresswell, Z.; Liang, S.; Benally, O. J.; Low, T.; Wang, J.-P. Large Spin–Orbit Torque with Multi-Directional Spin Components in Ni_4W. *Adv. Mater.* **2025**, *37*, 2416763.
